# The expression of B7-H3 isoforms in newly diagnosed glioblastoma and recurrence and their functional role

**DOI:** 10.1186/s40478-021-01167-w

**Published:** 2021-04-01

**Authors:** Marina Digregorio, Natacha Coppieters, Arnaud Lombard, Paul Noel Lumapat, Felix Scholtes, Bernard Rogister

**Affiliations:** 1 Laboratory of Nervous System Disorders and Therapy, GIGA-Neurosciences Research Centre, University of Liège, Avenue de L’Hôpital, 1, 4000 Liège, Belgium; 2 Department of Neurosurgery, CHU of Liège, Liège, Belgium; 3 Department of Neurology, CHU of Liège, Liège, Belgium

**Keywords:** Glioblastoma, B7-H3, Isoforms, Recurrence

## Abstract

Short survival of glioblastoma (GBM) patients is due to systematic tumor recurrence. Our laboratory identified a GBM cell subpopulation able to leave the tumor mass (TM) and invade the subventricular zone (SVZ-GBM cells). SVZ-GBM cells escape treatment and appear to contribute to GBM recurrence. This study aims to identify proteins specifically expressed by SVZ-GBM cells and to define their role(s) in GBM aggressiveness and recurrence. The proteome was compared between GBM cells located in the initial TM and SVZ-GBM cells using mass spectrometry. Among differentially expressed proteins, we confirmed B7-H3 by western blot (WB) and quantitative RT-PCR. B7-H3 expression was compared by immunohistochemistry and WB (including expression of its isoforms) between human GBM (*N* = 14) and non-cancerous brain tissue (*N* = 8), as well as newly diagnosed GBM and patient-matched recurrences (*N* = 11). Finally, the expression of B7-H3 was modulated with short hairpin RNA and/or over-expression vectors to determine its functional role in GBM using in vitro assays and a xenograft mouse model of GBM. B7-H3 was a marker for SVZ-GBM cells. It was also increased in human GBM pericytes, myeloid cells and neoplastic cells. B7-H3 inhibition in GBM cells reduced their tumorigenicity. Out of the two B7-H3 isoforms, only 2IgB7-H3 was detected in non-cancerous brain tissue, whereas 4IgB7-H3 was specific for GBM. 2IgB7-H3 expression was higher in GBM recurrences and increased resistance to temozolomide-mediated apoptosis. To conclude, 4IgB7-H3 is an interesting candidate for GBM targeted therapies, while 2IgB7-H3 could be involved in recurrence through resistance to chemotherapy.

**Supplementary Information:**

The online version contains supplementary material available at 10.1186/s40478-021-01167-w.

## Introduction

Glioblastoma (GBM) is the most common and lethal primary brain tumor in adults with short median survival (15 months) despite modern treatment: surgery followed by radio- and chemotherapy [26]. This disastrous mean survival time is due to systematic GBM recurrence [39]. Current literature suggests a role for GBM stem cells (GSC) in GBM initiation, maintenance and resistance to therapy. This makes them potential cells of origin for GBM recurrence.

GSC identification remains challenging. As already published by others, a single marker is not sufficient to specifically identify GSC [9, 31]. Our laboratory previously identified a GBM cell subpopulation able to leave the tumor mass (TM, “TM-GBM cells”) and invade the subventricular zone (SVZ, “SVZ-GBM cells”), a neurogenic niche in the adult brain, following xenotransplantation. SVZ-GBM cells express neural stem cell (NSC) markers and are more tumorigenic than TM-GBM cells [10, 16]. Thus, SVZ-GBM cells can be considered as a tumor cell subpopulation enriched with “aggressive” features. To better characterize this population of GBM cells, we compared the proteome of TM-GBM cells with that of SVZ-GBM cells, and identified a differentially expressed protein belonging to the B7-family members of immune checkpoints, B7-H3.

Human B7-H3 is encoded by *CD276.* It is a type I transmembrane protein with an extracellular part containing a repetition of IgV- and IgC- like domains in humans [19]. Two distinct B7-H3 isoforms, 4IgB7-H3 (~ 100 kDa) and 2IgB7-H3 (~ 50 kDa), are produced by alternative splicing [19, 25]. Although B7-H3 mRNA is ubiquitous, its protein expression is restricted to resting fibroblasts, endothelial cells, osteoblasts, activated T lymphocytes, Natural Killer (NK) and Antigen Presenting Cells (APC) [3, 28]. The B7-H3 receptor identity remains unknown; however, several studies have identified a potential receptor expressed on activated T cells [3, 28] and monocytes/macrophages [20]. B7-H3 is overexpressed in many types of cancers including GBM where it has been associated with tumor aggressiveness and poor prognosis [33, 37, 40, 42]. Once expressed by tumor cells, B7-H3 can act as an immune checkpoint favoring tumor-immune escape [17, 36]. Apart from a role in immune-modulation, B7-H3 confers a more aggressive phenotype to tumor cells [5, 8, 13, 36, 42, 44]. Once expressed by tumor cells, B7-H3 can therefore confer features to these cells through what we will systematically named “intrinsic” functional role of B7-H3.

In this study, we compared the expression of both B7-H3 isoforms between human GBM and non-cancerous brain tissue and between newly diagnosed and patient-matched recurrences. Cell types expressing B7-H3 in human GBM tissue were identified. Finally, B7-H3 expression was modulated with short hairpin RNA (shRNA) and/or over-expression (OE) vectors, and “intrinsic” features were studied in vitro with functional assays and in vivo with a xenograft mouse model of GBM.

## Methods

### Public mRNA expression databases

Data for the gene expression study were obtained from REpository for Molecular BRAin Neoplasia DaTa (REMBRANDT), The Cancer Genome Atlas (TCGA) and Chinese Glioma Genome Atlas (CGGA) databases using the GlioVis data portal, an online data visualization and analysis tool. The information (age, sex, *IDH* status and glioma grade) for patients analyzed in databases are available in Additional File 1: Table S1. *CD276* gene expression profiles in different cell types was assessed using data generated by Darmanis et al. [7] which represent Single Cell RNA-Seq Gene Expression of 3 589 cells from GBM. Cell types were specifically isolated based on biomarkers expression: SOX9 and EGFR for neoplastic cells, CD45 for myeloid cells, O4 for oligodendrocytes, CD90 for neuronal cells, HepaCAM for astrocytes and BSL-1 finally allowed endothelial cells isolation. Database is available in: http://gbmseq.org/. Details are given in Additional File 1.

### Human tissues

Human GBM samples were obtained in collaboration with the Neurosurgical department of the academic hospital (CHU of Liège, Liège, Belgium) in accordance with the research protocol (Belgian number: B707201420125) with approval from the ethical committee of the CHU of Liège. Additional GBM and non-cancerous brain tissues from donors were obtained from the Biobank of the Hospital and the University of Liège (BHUL, Uliège, Liège, Belgium) according to the protocol approved on the 12th of July 2016 by the Ethical Committee of the CHU of Liège. All patients gave informed consent before the study started. All tissues were examined by a board-certified neuropathologist. Human brain tissues include formalin-fixed paraffin-embedded (FFPE) for immunohistochemistry (IHC) (Additional File 1: Table S2), fresh frozen for western blot (WB) analysis (Additional File 1: Table S3 and S4), or tissues freshly dissociated to establish primary cell culture (Additional File 1: Table S5). Supplementary Tables are available in Additional File 1.

### Cell culture and primary GBM cell isolation

U87MG human GBM cells were purchased from Sigma-Aldrich. GB138 GBM cells were established in 2011 from a resected adult GBM sample obtained through our collaboration with the Neurosurgical department of the hospital (CHU of Liège) [16]. Other primary GBM cell cultures (T08 and T018) were isolated and dissociated as previously described [23]. Details about cell culture are given in Additional File 1: Table S5.

### Lentiviral transduction

U87MG and GB138 cells were stably transduced with pLV[shRNA]-mCherry:T2A:Bsd-U6 > {shB7-H3} or {shNT} (Additional File 1: Table S6), and pLV[Exp]-Neo-EF1A > CD276:IRES:EGFP or pLV[Exp]-Neo-EF1A > EGFP for the control vector (Additional File 1: Table S7). Details about sequences used to decrease or over-express *CD276* are described in Additional File 1: Table S6 and S7 and vector maps are shown in Additional File 2: Fig. S6.

### Intra-striatal transplantation, animal perfusion and human GBM cell isolation following xenograft

Adult (P40) female *Nu/Nu Nude* (immuno-deficient) mice (Crl:NU-*Foxn1*^*nu*^) obtained from Charles River Laboratories® (Wilmington, UK) were cared for in accordance with the Declaration of Helsinki, following the guidelines of the Belgium Ministry of Agriculture in agreement with the European Commission Laboratory Animal Care and Use Regulation (86/609/CEE, CE of J nL358, 18 December 1986). Animals were housed in sterilized filter-topped cages with unlimited access to water and food. Up to five mice were housed per cage, all cages numbered and labelled, and animals were handled as approved by the ethical committee of the University of Liège (Uliège, Liège, Belgium). Intra-striatal transplantation, animals perfusion and brain sections as well as human GBM cells isolation were performed as previously described [16]. Detailed procedures can be found in Additional file 1. Tumor volume was calculated on GFP positive areas in coronal brain sections using the following formula: Volume = 0.5 × length × width × height [14].

### Western blot

Proteins extracted from whole cell lysates were resolved with a 10% acrylamide/bis-acrylamide gel, transferred onto a PVDF membrane (Roche, Bâle, Swiss), and membranes were incubated with primary then secondary antibodies before being imaged using the ImageQuantTM LAS 4000 (GE Healthcare, Chicago, Illinois, USA). Detailed procedures, buffer composition and antibodies can be found in Additional file 1.

### Quantitative real-time polymerase chain reaction (qRT-PCR)

Total RNA was extracted using the TRIzol® (Invitrogen, Carlsbad, California, USA)—Chloroform method following the manufacturer’s instructions. Complementary DNA synthesis was performed following the Moloney Murine Leukemia Virus Reverse Transcriptase protocol (Promega, Madison, Wisconsin, USA). Finally, relative gene expression was quantified using Takyon No ROX SYBR 2X MasterMix blue dTTP (Eurogentec S.A, Liège, Belgium) and measured with LightCycler®480 Instrument (Roche). Primers sequences and data analysis can be found in Additional file 1.

### Immunostaining

Cells were fixed in 4% PFA for 10 min at room temperature, permeabilized with PBS-0.2%TritonX100 (PBS-T), incubated with primary antibodies (over-night (O/N) at 4 °C) then with fluorescently-labelled secondary antibodies (3 h at 4 °C). FFPE human brain tissue sections (5 μm-thick) were heated at 60 °C, dewaxed and rehydrated through a series of alcohol baths: 100% xylene (2 × 20 min), 100% ethanol (2 × 5 min), 95% ethanol, 80% ethanol, 75% ethanol and H_2_O (1 × 2 min each). If required, a heat-induced epitope retrieval step was performed using Tris–EDTA buffer (10 mM Tris base, 1 mM EDTA solution, 0.05% Tween 20, pH 9.0). Brain slices were heated in a pressure cooker (± 121 °C for 3 min) then were left to cool down for 2 h at room temperature before the immunostaining. Next, human FFPE or fixed frozen mouse brain sections (14 μm-thick) were permeabilized with PBS-T for 20 min, incubated 30 s with TrueBlack® Lipofuscin Autofluorescence Quencher (Biotium, Fremont, California, USA), blocked with PBS-10% donkey serum, then incubated with antibodies as described above. B7-H3 was co-labelled with markers specific for neoplastic cells (SOX2 and Nestin) harboring less differentiated states [7, 30], for endothelial cells (CD31) and pericytes (PDGFRβ) to stain blood vessels, and for macrophages/microglial cells (Iba1). Microglial cells were specifically identified using TMEM119 staining [2, 6, 35]. Nuclei were counterstained with a DAPI solution. Antibodies and data analysis can be found in Additional file 1.

### Apoptosis assay

The presence of apoptotic cells was assed using an Annexin V/DAPI staining protocol followed by FACS analysis following the manufacturer’s instructions (Fisher Scientific, Hampton, New Hampshire, USA). Details procedures can be found in Additional file 1.

### Statistical analysis

The GraphPad Prism software (version 5.03 for Windows, GraphPad Software, USA) was used for statistical analysis and for making graphs. The D'Agostino & Pearson omnibus normality test was used to control for normal distribution. Two-group means of values comparison was performed using unpaired Student *t*-test with Welsh correction if necessary. If more than two groups were compared, a One-way analysis of variance (ANOVA1) followed by a Bonferroni's Multiple Comparison Test was used for parametric analysis and if required Kruskal–Wallis test followed by Dunn's Multiple Comparison Test was performed for non-parametric analysis. A log-rank test was used to study the effect of a variable, between two groups, on life expectancy. The Hazard Ratio (HR) quantified the risk of death. A *p* value of ≤ 0.05 was considered as statistically significant.

## Results

### B7-H3 is increased in glioblastoma cells nested in the subventricular zone.

**Fig. 1:**
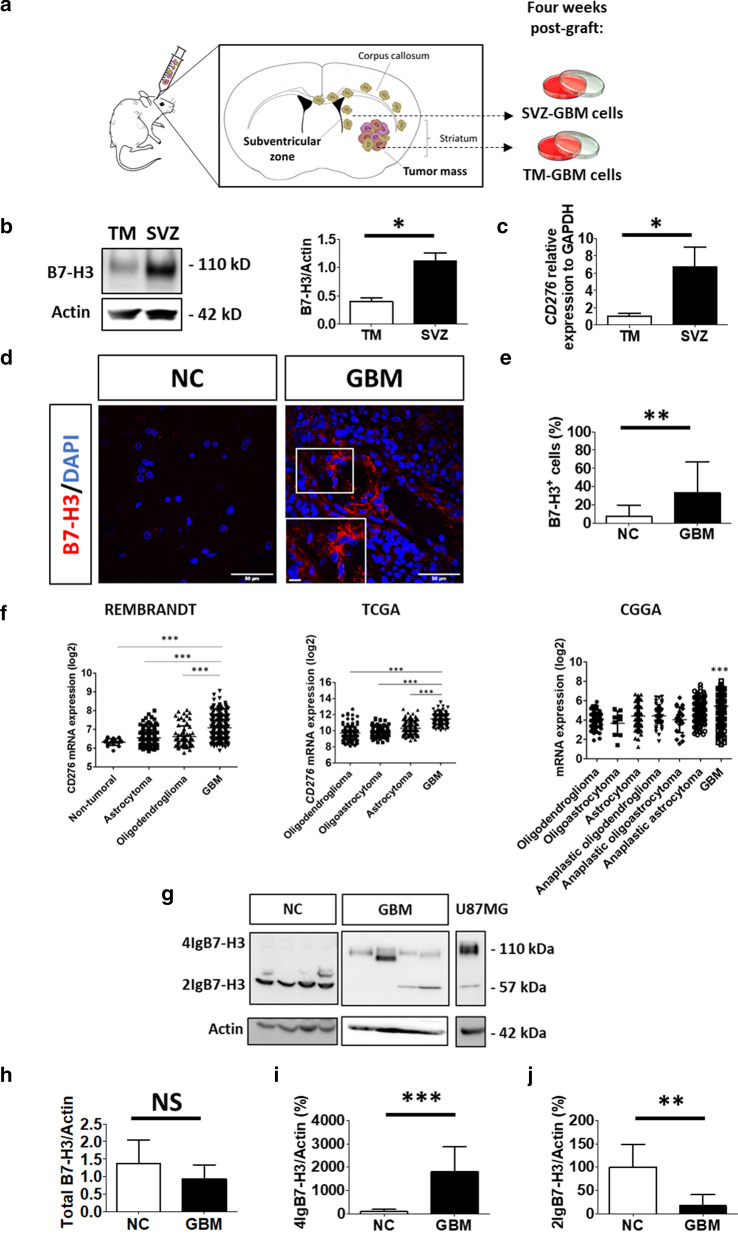
B7-H3 is increased in SVZ-GBM cells and in GBM tissue, with specific 4IgB7-H3 isoform expression. **a** Schematic representation of the xenograft mouse model. Four weeks post-graft, U87MG cells were isolated from the initial tumor mass (TM-GBM cells) or from the subventricular zone (SVZ-GBM cells) and established in culture. **b** Western blot (WB) analysis of B7-H3 in proteins extracted from TM- and SVZ-GBM cells (*N* = 3). **c** Quantitative RT-PCR analysis of B7-H3 normalized to GAPDH in cells described in B. (*N* = 3). **d** Immunofluorescent staining of B7-H3 (red) and nuclei (DAPI, blue) in non-cancerous (NC) brain (*N* = 5) *vs* GBM (*N* = 6) tissues. Scale bar = 50 µm and scale bar for enlarged images = 10 µm. **e** Immunofluorescent quantification showing the percentage of B7-H3 positive cells relative to the total number of cells (DAPI positive) in tissues described in (**d**). **f** *CD276* mRNA expression analysis from REpository for Molecular BRAin Neoplasia DaTa (REMBRANDT), The Cancer Genome Atlas (TCGA) and Chinese Glioma Genome Atlas (CGGA) databases in GBM (*N* = 219 REMBRANDT, *N* = 152 TCGA and *N* = 388 CGGA) compared to non-tumoral brain tissues (*N* = 28 REMBRANDT) and lower-grade gliomas; oligodendroglioma (*N* = 67 REMBRANDT, *N* = 191 TCGA and *N* = 112 CGGA), oligoastrocytoma (*N* = 130 TCGA and *N* = 9 CGGA), astrocytoma (*N* = 147 REMBRANDT, *N* = 134 TCGA and *N* = 175 CGGA), anaplastic oligodendroglioma (*N* = 94 CGGA), anaplastic oligoastrocytoma (*N* = 21 CGGA) and anaplastic astrocytoma (*N* = 214 CGGA). Data are given as mean ± SD *** < 0.001 (ANOVA1). **g** Representative image of a WB analysis of B7-H3 (4IgB7-H3; 2IgB7-H3). Four samples in each group are shown out of *N* = 8 for NC brain and *N* = 14 for GBM. U87MG cells were used as control. **h–j** WB quantification of total B7-H3 (4IgB7-H3 + 2IgB7-H3), 4IgB7-H3 or 2IgB7-H3, respectively. Quantification was based on *N* = 8 for NC brain and *N* = 14 for GBM. Actin was used as internal control for WB analysis. Normalized quantification is relative to NC brain tissue and expressed as a percentage. Graphs are mean ± SD with NS = not significant, **p < 0.01 and *** < 0.001 (*t*-test)

U87MG GBM cells were grafted in the right striatum of immunodeficient mice and allowed to migrate into the SVZ [16]. Four weeks post-graft, GBM cells were recovered from both the TM and the SVZ before being established in culture (Fig. 1a) and analyzed by mass spectrometry. Results demonstrated a significant increase in B7-H3 in SVZ-GBM cells compared to TM-GBM cells, which could be validated at protein and mRNA levels (Fig. 1b, c), confirming that B7-H3 is over-expressed in SVZ-GBM cells compared to TM-GBM cells.

### The expression of B7-H3 is altered in human glioblastoma.

For IHC, *N* = 6 GBM tissues were compared with *N* = 5 non-cancerous brain tissues (Additional file 1: Table S2). There is an increase in the number of B7-H3 positive cells in GBM in comparison to non-cancerous tissues (GBM: 33.3% B7-H3^+^ cells *vs* non-cancerous: 7.2% B7-H3^+^ cells) (Fig. 1d, e). In agreement, REMBRANDT, TCGA and CGGA database analysis revealed a higher level of mRNA coding for B7-H3 (*CD276*) in GBM compared to non-cancerous brain tissues or lower grades gliomas; oligodendroglioma, oligoastrocytoma and astrocytoma. In addition, in CGGA analysis, gliomas (WHO grade II) were compared with anaplastic gliomas (WHO grade III) and GBM (WHO grade IV) (Fig. 1f). For WB analysis, a new set of patients was investigated to study B7-H3 isoforms expression in GBM (*N* = 14) *vs* non-cancerous brain tissues (*N* = 8) (Additional File 1: Table S4). No change in total B7-H3 could be detected in GBM by WB (Fig. 1g, h). However, the study of each B7-H3 isoforms individually, revealed that 2IgB7-H3 was the only isoform present in the non-cancerous human brain and was decreased in GBM. Furthermore, 4IgB7-H3 was exclusively expressed in GBM (Fig. 1g, i, j). Interestingly, TCGA, CGGA and Rembrandt data analysis showed that *CD276* gene expression negatively predicted survival of patients with GBM or glioma (Additional file 2: Fig. S1).

### B7-H3 is expressed by neoplastic cells

**Fig. 2:**
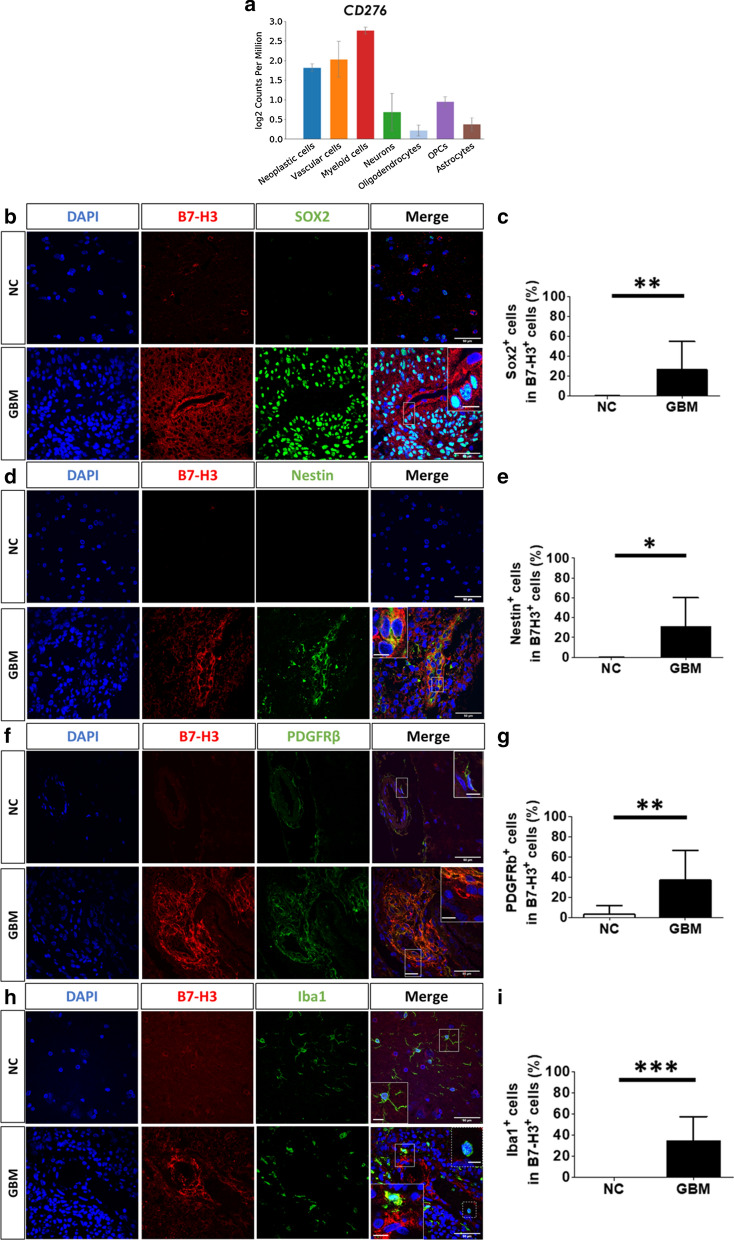
B7-H3 is mainly expressed by neoplastic cells, pericytes and myeloid cells. **a** *CD276* gene expression profiles in different cell types using data generated by Darmanis et al*.* (2017). **b**, **d**, **f**, and **h** Representative images of B7-H3 (red) co-labeled with (**b**) SOX2 (**d**) Nestin (**f**) PDGFRβ and (**h**) Iba1 (green) in non-cancerous (NC) brain (*N* = 5) versus GBM (*N* = 6) tissues. DAPI (blue) was used to counterstain nuclei. Major scale bar = 50 µm and scale bar for enlarged images = 10 µm. **c**, **e**, **g** and **i** Percentage of B7-H3 positive cells expressing SOX2, Nestin, PDGFRβ and Iba1 in NC brain (*N* = 5) versus GBM (*N* = 6) tissues. Graphs are mean ± SD *p < 0.05 **p < 0.01 and ***p < 0.001 (*t*-test)

B7-H3 was detected in the cytoplasm and/or membrane of cells. Staining was mainly detected around potential lumen of vessels (Fig. 1d) or in dense cellular areas with hyperchromatic nuclei. These histological features suggest that B7-H3 is mainly expressed in cells showing aggressive features in GBM tissues. We analyzed a database built on a single cell RNA-Seq gene expression analysis [7] of human GBM samples which showed that *CD276* mRNA is mainly expressed by neoplastic, vascular and myeloid cells (Fig. 2a). Next, the identity of cells expressing B7-H3 was confirmed through immunolabelling of human GBM and systematically compared with human non-cancerous brain tissues.

As expected, SOX2 and Nestin were detected in GBM but poorly expressed in non-cancerous brain tissues. Co-labelled experiments revealed that 26.4% and 35.1% of B7-H3 positive GBM cells expressed SOX2 or Nestin, respectively (Fig. 2b–e). Thus, over a quarter of cells highly positive for B7-H3 are cancerous cells with a stem cell profile.

The density of blood vessels was higher in GBM compared to non-cancerous brain tissues with pericytes being the main cells highly positive for B7-H3 (36.9% PDGFRβ^+^cells in B7-H3^+^ cells) (Fig. 2f, g). Surprisingly, the percentage of endothelial cells (CD31^+^) in B7-H3 positive cells did not differ between non-cancerous brain (6.25%) and GBM (3.7%) tissues (Additional file 2: Fig. S2A and B).

Finally, 34.5% of B7-H3^+^ cells were Iba1 positive, with 16.3% being microglia (TMEM119^+^) (Fig. 2h, i and Additional file 2: Fig. S2C and D). In the contrary, B7-H3 was not detected in macrophages nor in microglia from non-cancerous brain (Fig. 2h and Additional File 2: Fig. S2C) indicating that the expression of B7-H3 in these cells is exclusive to GBM specimens.

The number of B7-H3^+^ cells in a defined cell type was then counted in GBM (Additional file 2: Fig. S3). Approximately forty percent of SOX2 or Nestin positive cells were B7-H3 positive, with 44.4% and 35.2% respectively (Additional file 2: Fig. S3A and B). A quarter (26.2%) of endothelial cells (CD31^+^) was B7-H3 positive whereas the majority (65.1%) of pericytes (PDGFRβ^+^) expressed B7-H3 (Additional file 2: Fig. S3C and D). Finally, almost one third of macrophages and microglial cells expressed B7-H3 in GBM with 33% and 32.7% B7-H3 positive cells in Iba1 and TMEM119 positive cells respectively (Additional file 2: Fig. S3E and F).

### 2IgB7-H3 has no functional role in glioblastoma stem cell potential

**Fig. 3:**
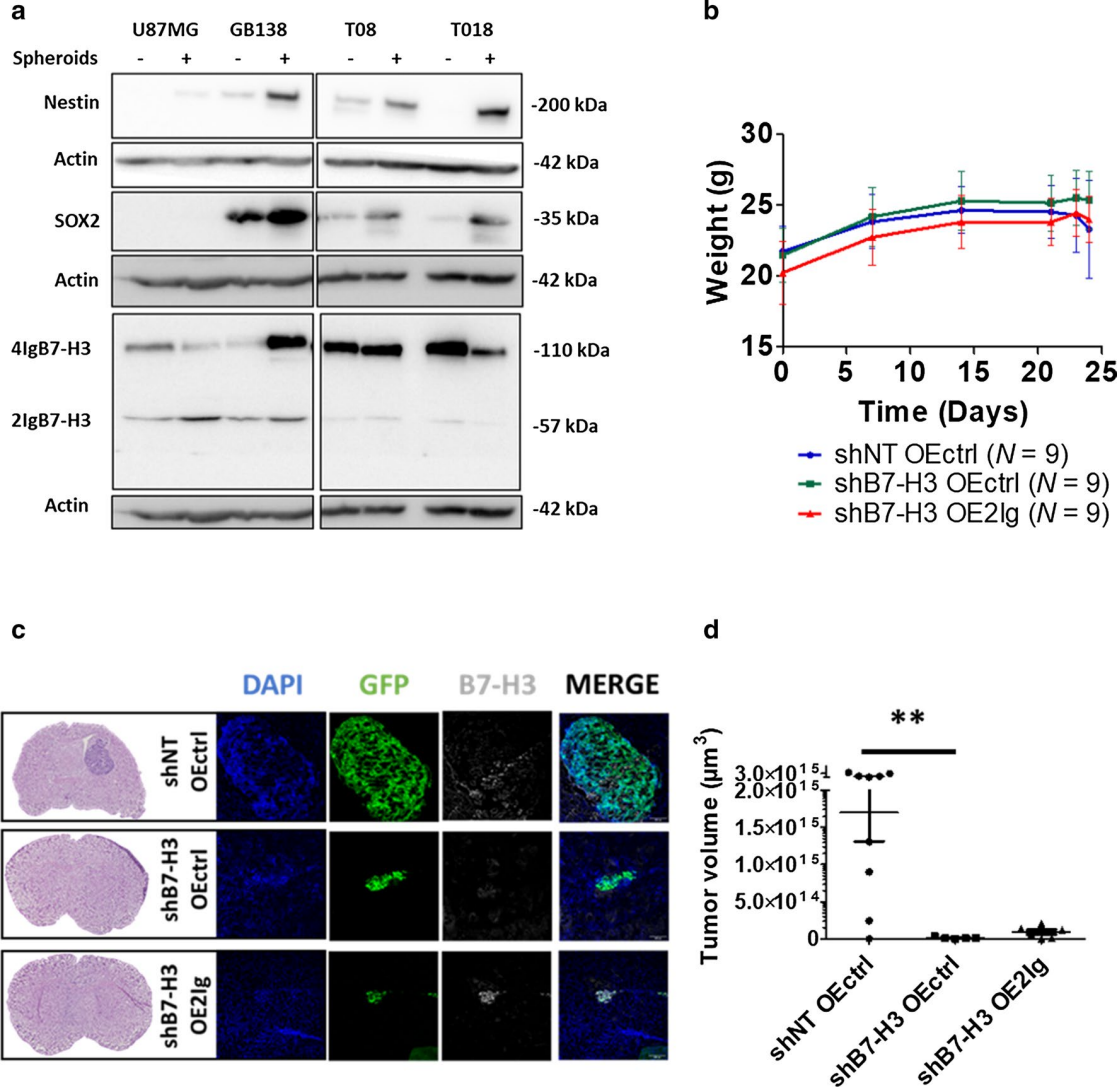
Decreased B7-H3 expression in human GBM cells impairs tumor growth in vivo. **a** Representative images of a western blot analysis of stemness markers, Nestin or SOX2 as well as 4IgB7-H3 and 2IgB7-H3 in U87MG, GB138, T08 or T018 human GBM cells cultured as spheroids ( +) or in adherent conditions (−). **b** Graphs showing the weight of mice over time (days). *Nude* mice were grafted with U87MG human GBM cells transduced with shNT OEctrl (*N* = 9; blue line), shB7-H3 OEctrl (*N* = 9, green line) or shB7-H3 OE2Ig (*N* = 9, red line). **c** Left: Coronal sections of representative mouse brains grafted with cells described in (**b**). Sections are at the level of the lateral ventricles and are stained with Hematoxylin and Eosin. Right: Representative images of B7-H3 (Grey) in tumors (GFP-positive U87MG cells; green). Nuclei were counterstained with DAPI (blue). Scale bar = 50 µm. **d** Quantification of the tumor volumes (in µm^3^) in mice grafted as described in (**b**). Graphs are given with the mean ± SD, **p < 0.01 (ANOVA1)

As ~ 30% B7-H3 positive GBM cells are neoplastic cells harboring less differentiated states (SOX2^+^), we assessed whether B7-H3 (both isoforms) levels vary between differentiated states. Thus, GBM cells (cell line and primary GBM cells) were either cultured as spheroids (“3D”), under condition enhancing their stemness properties, or as monolayers (“2D”). As expected, SOX2 and Nestin increased in all cultures investigated when placed in 3D conditions. In addition, 2IgB7-H3 increased in three of the studied human GBM cultures, with the opposite results obtained for T018. No conclusion could be drawn for 4IgB7-H3 expression using the same samples (Fig. 3a). This experiment suggests that 2IgB7-H3 is mainly expressed by GBM cells harboring a lesser differentiated state.

Then, human GBM cells were transduced with five different B7-H3 short hairpin RNA (shRNA) to decrease the expression of B7-H3. GBM cells transduced with shRNA non-target (shNT) were used as control. Among the five B7-H3 shRNA tested, we continued our experiments with shRNA #5 and #3 for GB138 and U87MG GBM cells respectively, as they were the most efficient at decreasing B7-H3 expression in these cells. Finally, as 2IgB7-H3 expression was increased in GBM cells cultured in conditions favoring self-renewal capacities, 2IgB7-H3 isoform was over-expressed (OE) in the different cell types. After validation by WB and immunofluorescence assays of the modulation of B7-H3 expression using shRNA and/or OE vector (Additional file 2; Fig. S4A, B, and C), we determined B7-H3 “intrinsic” functional role in human GBM. The study of the functional role of 4IgB7-H3 specifically, was not possible since expressing the large isoform would automatically produce 2IgB7-H3 (Additional file 2; Fig. S4B).

Our results obtained from various assays revealed no direct role of B7-H3 in cell proliferation or cell viability (Additional file 2: Fig. S5A–E). However, the modulation of B7-H3 had significant effects when transduced GBM cells were grafted in the right striatum of adult (P40) immunodeficient (*Nude*) mice. Mice grafted with U87MG shNT OEctrl cells started to lose weight 23 days post-graft whereas mice grafted with U87MG shB7-H3 OEctrl cells maintained their body weight until the end of the experiment (Fig. 3b). Immunofluorescent staining confirmed the expression of B7-H3 in shNT OEctrl and shB7-H3 OE2Ig tumors and a reduction in its expression in U87MG shB7-H3 OEctrl tumors (Fig. 3c). A GFP positive tumoral mass was detected in *N* = 9/9, *N* = 5/9 and *N* = 6/9 mice grafted with U87MG shNT OEctrl, shB7-H3 OEctrl or shB7-H3 OE2Ig cells, respectively. The quantification of GFP positive areas from coronal brain sections showed a significant reduction in the tumor volume in mice grafted with U87MG shB7-H3 OEctrl cells compared to U87MG shNT OEctrl cells-grafted mice (Fig. 3c, d). However, the rescue experiment using shB7-H3 OE2Ig revealed that the expression of the 2IgB7-H3 isoform alone is not sufficient to reverse the phenotype (Fig. 3 c, d) and thus, does not confer tumorigenicity to GBM cells.

### 2IgB7-H3 increases in glioblastoma recurrence and confers resistance to temozolomide

**Fig. 4:**
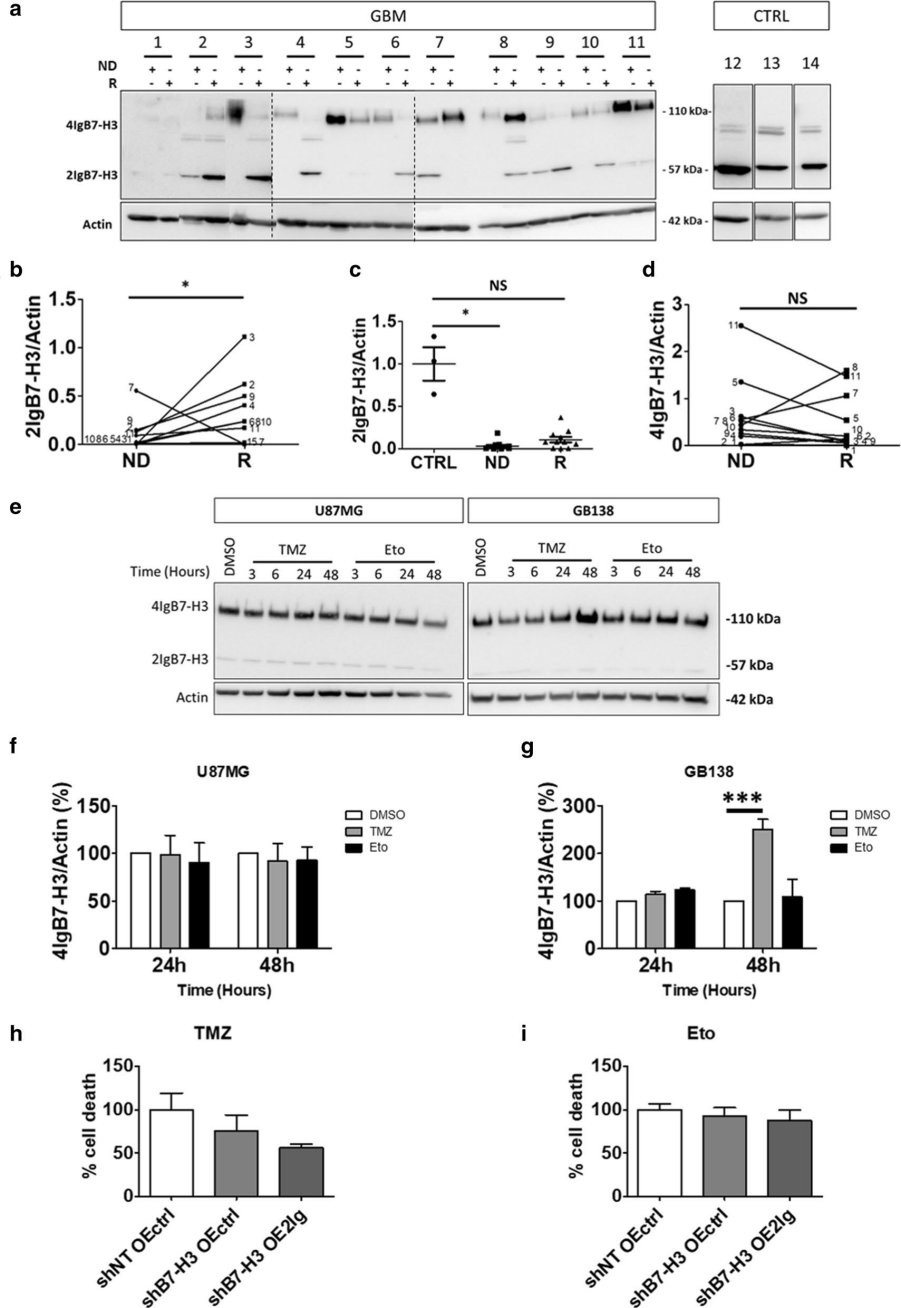
2IgB7-H3 increases in GBM recurrence and confers resistance to temozolomide. **a** Representative image of a western blot (WB) analysis of 4IgB7-H3 and 2IgB7-H3 in newly diagnosed GBM (ND, *N* = 11), in patient-matched recurrence (R), and in non-tumoral patients (CTRL, *N* = 3, 12 to 14). Vertical dashed lines delimit each gel. **b–d** WB quantification of 2IgB7-H3 or 4IgB7-H3 in ND *vs* R. Dots represent the values obtained for each of the variables in specific condition (ND *vs* R) and are annotated with respective patient number (**b** and **d**). ND and R tissues were also compared with CTRL tissues with graphs representing mean ± SD (**c**). NS = not significant and *p < 0.05 (*t*-test for **b** and **d** and ANOVA1 for **c**). **e **Representative images of a WB analysis of 4IgB7-H3 and 2IgB7-H3 in U87MG or GB138 cells treated with 250 µM TMZ, 20 µM etoposide (Eto) or vehicle (DMSO) during 3, 6, 24 or 48 h (*N* = 3). **f**, **g** WB quantification of 4IgB7-H3 expression respectively in U87MG or GB138 cells treated as described in (**e**). Normalized quantification is relative to DMSO condition. **h**, **i** Graphs quantifying cell death using AnnexinV/DAPI staining protocol followed by FACS in GB138 cells transduced with short hairpin RNA (shRNA) against B7-H3 (shB7-H3) or non-target (shNT) and vector over-expressing (OE) 2IgB7-H3 (OE2Ig) or control (OEctrl) after a 48-h exposure to TMZ or Eto, respectively. The percentage of cell death was relative to shNT OEctrl condition and expressed as a percentage. Actin was used as internal control for WB analysis. Graphs represent mean ± SD and are representative of three independent experiments (*N* = 3). ***p < 0.001 (ANOVA1)

A new set of patients was enrolled to study B7-H3 (both isoforms) expression between human newly diagnosed GBM samples and their respective recurrence (*N* = 11). All patients have received the same treatment scheme, namely the Stupp’s protocol (Additional file 1; Table S3) [26]. Non-cancerous brain tissues were used as control tissues (CTRL, *N* = 3) (Fig. 4a). Note that non-cancerous brain tissues express a third band for B7-H3 (~ 70 kDa). This could correspond to a B7-H3 fragment remaining in the membrane after cleavage and release of soluble B7-H3 (30 kDa) [41]. 2IgB7-H3 expression increased in most recurrences compared to their respective newly diagnosed GBM (Fig. 4b). Moreover, when newly diagnosed GBM or recurrent GBM were individually compared with CTRL tissues, decreased 2IgB7-H3 expression was only observed in newly diagnosed GBM compared to CTRL. Thus, the highest level of 2IgB7-H3 was found in non-GBM tissues and the lowest level was detected in newly diagnosed GBM. The expression of 2IgB7-H3 in GBM recurrences was higher than in newly diagnosed GBM without reaching the level of that detected in CTRL tissues (Fig. 4c). Out of the eleven tumors investigated, only three showed an increased in 4IgB7-H3 expression in recurrences. For one patient, there was no difference in 4IgB7-H3 expression and for the seven remaining patients there was a decrease in 4IgB7-H3 isoform in GBM recurrences. However, when all cases were gathered, there was no significant difference in 4IgB7-H3 expression between newly diagnosed GBM and recurrences (Fig. 4d). Taken together, these data show an enrichment of 2IgB7-H3 expression in recurrent GBM tissues, which could result from the treatments received by the patient. Indeed, increased chemo-resistance is one of the “intrinsic” functions of B7-H3 reported in various cancer types [8, 12, 29, 43]. To test this function in GBM, cells were cultivated for various lengths of time in the presence of two chemotherapeutic agents used for GBM treatment: Temozolomide (TMZ, 250 µM) or Etoposide (Eto, 20 µM). Treatment with TMZ during 48 h increased 2IgB7-H3 and 4IgB7-H3 expression in GB138 cells but not in U87MG cells. On the contrary, etoposide did not modulate B7-H3 expression in U87MG nor GB138 cells for any of the time points (Fig. 4e–g). Since B7-H3 expression was only modified in GB138 cells following TMZ treatment, B7-H3 role in apoptosis resistance was only analyzed in these cells. To show that apoptosis was specifically due to TMZ, we compared TMZ-treated GBM cells with cells treated with Eto treatment. Apoptosis was assessed using an Annexin V/DAPI staining protocol followed by FACS analysis. Cells expressing only the 2IgB7-H3 isoform were more resistant to TMZ with a 50% decrease of apoptosis. B7-H3 knock down (shB7-H3 OEctrl) did not modify resistance to TMZ-induced apoptosis compared with shNT OEctrl (Fig. 4h). Finally, B7-H3 expression did not modify resistance to Eto.-induced GB138 apoptosis (Fig. 4i).

## Discussion

Despite multimodal therapy, GBM systematically relapse due to remaining cancerous cells. We previously demonstrated the existence of a population of GBM cells enriched in GSC and able to migrate out of the tumor mass, infiltrate the surrounding parenchyma and migrate to the SVZ where they find a “hideout” [10, 16]. Once nested in the SVZ, these GBM cells escape a surgical intervention and are more resistant to radiotherapy. This suggests their contribution to tumor recurrence [11]. The present study showed that B7-H3 was increased in GBM cells located in the SVZ, compared to those in the TM. B7-H3 has previously been associated with malignant processes such as cell proliferation and angiogenesis [37].

Higher B7-H3 expression in GBM, compared to lower grade gliomas, suggests a role of B7-H3 in glioma aggressivity [17, 37, 42]. In our study, B7-H3 expression was increased both at the protein and the transcriptional level in GBM compared to non-cancerous brain tissue. In agreement, Zhang and colleagues recently reported that B7-H3 protein expression was higher in GBM tissues compared to non-cancerous adjacent tissue [42]. Analysis of the CGGA and TCGA databases, showing hypo-methylation of the B7-H3 gene promotor in GBM, supports the hypothesis of transcriptional regulation of B7-H3 in GBM [40].

In humans, two B7-H3 isoforms, known as 4IgB7-H3 and 2IgB7-H3, are found. They are produced by a *CD276* mRNA alternative splicing [19, 27]. The present study is the first to specifically compare the expression of B7-H3 isoforms between human GBM and non-cancerous brain tissues through WB analysis. B7-H3 is an ubiquitous protein which is expressed at relatively low level in the adult non-cancerous brain [32]. The expression of each B7-H3 isoforms vary across tissue types with 4IgB7-H3 being the major B7-H3 isoform expressed in most tissues, including skeletal muscle, kidney, pancreas and heart tissues. In the brain, however, both isoforms are expressed at similar levels, 4IgB7-H3 being massively decreased compared to other tissues [28]. In our investigation, 2IgB7-H3 was even the only B7-H3 isoform expressed in non-cancerous brain tissue. Only in GBM tissue, a significant amount of the 4IgB7-H3 isoform was detected (*N* = 14). This contradicts a study from Wang and colleagues where the 2IgB7-H3 isoform was specific to gliomas (32 positive glioma tissues out of 35) compared to normal brain tissues (0 positive normal brain tissues out of 10) [38]. However, in the latter investigation, patients and samples were not stratified according to glioma grade. Since the level of B7-H3 expression substantially varies among glioma grades, this might explain the discrepancy with our findings [17, 37, 42]. To be comparable to our analysis, 2IgB7-H3 and 4IgB7-H3 would have to be considered separately and analyzed in each grade of glioma. The observations made in the above mentioned results from Wang and colleagues compared to our results, could therefore suggest that 4IgB7-H3 protein expression is specific for GBM tissue.

B7-H3 can be detected in the cytoplasm and at the cell membrane [17]. This was confirmed in our study. In addition, we showed that, B7-H3 was mainly expressed by pericytes and macrophages specifically in GBM tissue. In pericytes, B7-H3 expression could be of interest. This cell type already demonstrated immunosuppressive effects when they interact with GBM cells, favoring GBM growth in vitro and in vivo [34]. As for macrophages, several studies have already related their expression to GBM cell proliferation, migration and immunosuppressive function, together promoting GBM growth [21, 22, 24]. The expression of B7-H3 by GBM macrophages leads to the inhibition of T cell-mediated immune response [1, 4]. However, the role of B7-H3 expressed by macrophages or pericytes in the modulation of neoplastic cell aggressiveness remains to be elucidated.

Endothelial cells from the microvascular of human non-cancerous brain express B7-H3 mRNA [17]. In the present investigation, B7-H3 was also detected in CD31^+^ GBM endothelial cells. There was, however, no significant difference in the percentage of B7-H3^+^ cells expressing CD31 in GBM *vs* non-cancerous brain tissue. B7-H3 has also been detected in circulating endothelial cells (CEC) and was overexpressed in the peripheral blood of patients with GBM (*N* = 83) than in control patients without a known tumor (*N* = 24) [15]. Since it appears specific to the presence of the tumor in GBM patients, the expression of B7-H3 by CEC, rather than the expression of B7-H3 in endothelial cells in the tumor tissue, may be considered for investigation as an indirect, non-invasive biomarker for GBM [15].

B7-H3 was also mainly expressed by SOX2 or Nestin positive cells that can be considered neoplastic [7]. Since SVZ-GBM cells, having a more “aggressive” phenotype [10, 16], also over-expressed B7-H3, we focused our investigation on the role of B7-H3 in neoplastic progression and tumor features. Although B7-H3 has been correlated with mitotic pathways in gliomas [37], our in vitro results did not confirm a role for B7-H3 in GBM cell survival or in DNA replication associated with cell division. These observations are in line with other studies which could not demonstrate that B7-H3 expression was associated with cancerous cell division or viability in vitro [5, 18, 42]. However, although B7-H3 was not associated with cell proliferation in vitro, its suppression in GBM cells significantly impaired tumor formation following orthotopic implantation in the present study. B7-H3 expression by GBM cells could thus play a role in tumor growth once in contact with the brain microenvironment, including macrophages and pericytes. In line with this hypothesis, a presumed B7-H3 receptor has already been detected on activated myeloid cells. This suggests a role for GBM cells-expressing B7-H3 on macrophage function and subsequent tumor progression [20].

Finally, although 2IgB7-H3 expression alone was not sufficient to rescue tumorigenicity, our results indicate that it might be involved in tumor recurrence. Indeed, 2IgB7-H3 expression was higher in recurrences compared to newly diagnosed human GBM tissues. Our in vitro experiment also showed a 50% decrease of TMZ-mediated apoptosis in GB138 cells over-expressing 2IgB7-H3 compared to control. A role of B7-H3 in chemo-resistance has been demonstrated for various other tumor types [8, 29, 43]. Moreover, JAK/STAT pathway was proposed as a potentially responsible for B7-H3-mediated TMZ resistance in GBM cells [45].

## Conclusion

These results reveal a potential importance of B7-H3 in GBM biology. In particular, we showed that the 2IgB7-H3 isoform is linked to a more aggressive phenotype of GBM cells and could therefore contribute to subsequent recurrence. This hypothesis is reinforced by the higher level of 2IgB7-H3 expression shown in recurrences compared to newly diagnosed human GBM tissues. This study, compared with the literature, also suggests that the 4IgB7-H3 isoform is specific for GBM tissue and would therefore be a good candidate for targeted molecular GBM therapy.

## Availability of data and material

The datasets supporting the conclusions of this article are included within the article (and its additional files). Concerning databases, data for the gene expression study were obtained from REpository for Molecular BRAin Neoplasia DaTa (REMBRANDT), The Cancer Genome Atlas (TCGA) and Chinese Glioma Genome Atlas (CGGA) databases using the GlioVis data portal, an online data visualization and analysis tool: https://gliovis.shinyapps.io/GlioVis/.

## Supplementary Information

**Additional file 1.** Additional_file_1_materials_Acta_Neuropath._Comm._review. This file contains additional information about materials and methods as well as Supplementary Tables S1–S7.

**Additional file 2.** Additional_file_2_figures_Acta_Neuropath._Comm_review. This file contains Supplementary Figures and legends S1–S6.

## Abbreviations

APC: Antigen-Presenting Cell
CGGA: Chinese Glioma Genome Atlas Database
CTRL: Control
Eto: Etoposide
FFPE: Formalin-Fixed Paraffin-Embedded
GBM: Glioblastoma Multiforme
GSC: Glioblastoma Stem Cell
IHC: Immunohistochemistry
NC: Non-cancerous
NK: Natural Killer Cell
NSC: Neural Stem Cell
NT: Non-Target
OE: Overexpression
qRT-PCR: Quantitative real-time polymerase chain reaction
REMBRANDT: REpository for Molecular BRAin Neoplasia DaTa database
shRNA: Short Hairpin Ribonucleic Acid
SVZ: Subventricular Zone
TCGA: The Cancer Genome Atlas database
TM: Tumor Mass
TMZ: Temozolomide
WB: Western Blot
