# Supplementary material for: The expression of B7-H3 isoforms in newly diagnosed glioblastoma and recurrence and their functional role
Source: Acta Neuropathol Commun. 2021 Apr 1;9:59. doi: 10.1186/s40478-021-01167-w (PMC8017683; doi:10.1186/s40478-021-01167-w)
Supplement: Supplementary file 1 — Additional file 1. Additional_file_1_materials_Acta_Neuropath._Comm._review. This file contains additional information about materials and methods as well as Supplementary Tables S1–S7. [file 40478_2021_1167_MOESM1_ESM.docx]

# Additional file 1

## Public mRNA expression databases

B7-H3 mRNA expression was compared between non-tumoral (*N* = 8), Oligodendroglioma (*N* = 67), Astrocytoma (*N* = 147) and GBM (*N* = 219) tissues from the REpository for Molecular BRAin Neoplasia DaTa (REMBRANDT) database. Survival plot data were obtained using The Cancer Genome Atlas (TCGA) database in GBM patients (*N* = 76 for both low- and high- *CD276* gene expressing tumors) or in GBM combined with low-grade glioma patients (LGG, *N* = 335 and *N* = 332 for patients whose tumors express high and low level of mRNA coding for *CD276,* respectively). Data were analyzed using the GlioVis data portalhttps://gliovis.shinyapps.io/GlioVis, an online data visualization and analysis tool.

## Cell culture condition

Cells were maintained in a 5% CO_2_ humidified incubator at 37°C. The medium used for “*2D*” culture (complete medium) is composed of Dulbecco's Modified Eagle Medium (DMEM) supplemented with glucose and L-glutamine (Lonza), 10% Heat Inactivated Fetal Bovine Serum (HI-FBS), 1% Penicillin-Streptomycin (PS; ThermoFisher scientific) and 0.2% Normocin (Invivogen). Complete medium confers an adherent phenotype to cells which consequently grow as monolayers. The medium used for “3D” cultures (stem cell media) is composed of DMEM/F12 (ThermoFisher scientific) containing 1x B27 without vitamin A (Fisher Scientific), 20 ng/ml human recombinant fibroblast growth factor 2 (hFGF-2, PeproTech), 20 ng/ml human recombinant epidermal growth factor (hEGF, PeproTech), 2 µg/ml heparin (LEO), 1% PS and 0.2% Normocin. Stem medium enables cells to form spheres and to grow in suspension (non-adherent cell growth). Fresh hFGF-2 and hEGF were added every second day to the stem cell medium to maintain cells in suspension and foster spheroid formation. Mycoplasma tests were performed on a regular basis for all cultures.

## Primary GBM cells isolation

After tumor resection, GBM samples were immediately transferred (< 5 min) to our laboratory. The tumor mass was first washed with iced cold Hank’s Balanced Salt Solution without calcium, magnesium and phenol red (HBSS, ThermoFisher scientific) and blood vessels as well as necrosis parts were dissected out. After cutting the tumor mass into small pieces (< 1 mm^3^), cells were dissociated using a solution containing Hibernate^TM^- A medium (ThermoFisher scientific), 10 U/ml Deoxyribonuclease I from bovine pancreas (DNase I, Sigma-Aldrich) and 2.5 U/ml Papaïn (PAP2, Worthington) followed by 20 min incubation at 37°C with gentle agitation. DMEM/F12 (ThermoFisher scientific) supplemented with 1x B27 without vitamin A (Fisher Scientific) was then added to the mix to dilute and thus stop the enzymatic activity before the cell suspension was filtered through a 70 µm cell strainer (Life Sciences) to remove debris. After a 10 min centrifugation at 160 x g, cells were resuspended in stem cell medium. Once cells in stem cell medium formed spheres, they were dissociated with Accutase (StemCell Technologies) for various experiments or preserved in Bambanker (Nippon Genetics Europe GmbH), a serum free freezing medium. Primary cell culture isolated from a patient was given a number which follows the order in which samples were received. This number was preceded by the letter “T” for cells isolated in stem cell media. Our experiments were performed on T08 and T018 GBM primary cells isolated in our laboratory. Information about patients for T08 and T018 are given in **Additional file 1: Table S5**.

## Lentiviral transduction

Cells were transduced by lentiviral vectors in collaboration with the GIGA Viral Vectors Platform (Uliège, Liège, Belgium). U87MG and GB138 were first transduced with five lentiviral vectors expressing short hairpin B7-H3 (shB7-H3) targeting both the 2Ig and the 4Ig B7-H3 isoforms or a non-target shRNA (shNT) for controls. Vectors were composed as followed; pLV[shRNA]-mCherry:T2A:Bsd-U6>{shB7-H3} or {shNT}. shRNA sequences are shown in **Additional file 1: Table S6.** These shRNAs were designed to target the 3’UTR end of the mRNA to enable rescue experiments. Cells efficiently transduced were selected using 8 µg/ml and 13 µg/ml of blasticidin (InvivoGen) for U87MG and GB138, respectively. U87MG and GB138 human GBM cells, were also transduced with lentiviral vectors overexpressing human the 2IgB7-H3 isoform or a control vector. Vectors were composed as followed; pLV[Exp]-Neo-EF1A>CD276:IRES:EGFP or pLV[Exp]-Neo-EF1A>EGFP for the control vector. mRNA sequence for human 2IgB7-H3 is shown in **Additional file 1: Table S7**. Cells efficiently transduced were selected using G418 (Geneticin) (InvivoGen): 2 mg/ml for U87MG and 1 mg/ml for GB138. Vector maps for shRNA, OE2IgB7-H3 and OE4IgB7-H3 are shown in **Additional file 2: Fig. S6 A.**, **B.** and **C.**, respectively.

## Intra-striatal transplantation, animal perfusion and human GBM cell isolation following xenograft

Intra-striatal transplantation was performed as previously described by our group ^1^. Briefly, mice were anesthetized with an intra-peritoneal injection of 10/80 mg/Kg of Xylazine/Ketamine. Anesthetized mice were placed into a stereotactic frame, the cranium was exposed after incision and the bregma (intersection of the coronal and sagittal sutures) was located and used as landmark. The coordinates used to drill a hole and transplant cells by injection, were 0.5 mm anterior and 2 mm to the right of the bregma, at a depth of 2 mm. 50 000 U87MG cells diluted in 2 µl of PBS were injected using a Hamilton Syringe (Hamilton Company). In accordance with the ethical committee of the University of Liège (Uliège, Liège, Belgium), all mice were sacrificed when any sign of suffering was visible or if a 20% weight-loss was observed for at least one mouse.

***For brain slices***: At day 23 post-graft, first signs of suffering and weight loss appeared in mice grafted with U87MG shNT OEctrl cells and all group of mice were sacrificed. 400 mg/kg of Euthasol vet (Kela) was injected intra-peritoneally to irreversibly anaesthetized animals. Mice were first perfused with a physiological saline (0.9% NaCl) solution to wash off the blood, followed by 4% paraformaldehyde (PFA) to fix tissues. Brains were removed then post-fixed in 4% PFA, overnight at 4°C and were kept in PBS-0.1% azide at 4°C. Before being cut serially into 14 μm-thick transverse (coronal) sections on a cryostat, mice brain fixed as described above were immersed in a PBS-30% sucrose solution allowing water removal from brains and their cryo-preservation. Next, slices were dried at room temperature before being stored at -80°C for subsequent immunofluorescence staining.

***For cells cultur*e**: after xenotransplantation of U87MG cells, mice were housed during four weeks to allow tumor growth and GBM cell migration into the subventricular zone (SVZ) as previously described ^1^. After four weeks, U87MG were micro-dissected from the initial tumor mass (TM) or from the SVZ where they have migrated. These two U87MG populations (U87MG-TM and U87MG-SVZ) were obtained from 300 µm-thick sections using a Leica Vibratome (Leica VT1000S). Micro-dissected tissue sections were dissociated into single cell suspension after incubation with papain for 30 min at 37°C then ovomucoïd (Worthington®) was added to inhibit its enzymatic activity. Dissociated U87MG-TM and U87MG-SVZ cells were plated in six-well plates in complete medium and were selected within one passage and immediately frozen or used for subsequent experiments. In total, three U87MG-TM and U87MG-SVZ cell populations were isolated from three independent mice and were put back in culture.

## Mass spectrometry (LC-MS/MS)

Total protein extractions were performed with 10 mM Tris-HCl pH7.4 containing 1X Halt^TM^ Protease and Phosphatase Inhibitor Cocktail EDTA-free (ThermoFisher Scientific) and 4% SDS. After protein quantification using RCDC kit (Biorad), LC-MS/MS was performed in collaboration with the GIGA Proteomics Platform (Uliège, Liège, Belgium). Briefly, 20 µg of proteins were reduced using dithiothreitol (DTT), alkylated with iodoacetamide and precipitated using 2D clean up kit (GE Healthcare). The protein pellets were further resolubilized in bicarbonate ammonium 50mM and digested with trypsin (Pierce MS Grade). For each sample, 5 µg of protein digest was purified on a Ziptip C18 (Millipore) according to the manufacturer’s instructions, dried and re-suspended in 100 mM Ammonium formate (pH 10) at 0.278 µg/µL. A standard MassPREP™ digestion mixture (Waters Corp.) was spiked in each sample at a quantity of 150 fmoles of ADH digest per injection. This commercial standard consists of two standard mixtures (MPDS Mix 1 and MPDS Mix 2) containing protein digests of Yeast Alcohol Dehydrogenase (ADH), Rabbit Glycogen Phosphorylase b, Bovine Serum Albumin and Yeast Enolase present at known protein ratio. All samples were injected on a 2D-nanoAquity UPLC (Waters, Corp., Milford, USA) coupled online with a Q Exactive Plus mass spectrometer (Thermo Scientific, USA) in nanoelectrospray positive ion mode (spray voltage at 2.2 kV, capillary temperature at 270°C, S-Lens RF level at 50). Briefly, the liquid chromatography approach used was a 2D--nanoUPLC system comprising three steps of 180 min. The samples were loaded at 2 μL/min (20 mM ammonium formate solution adjusted to pH 10) on the first column and subsequently eluted in three steps (13.3%, 19% and 65% acetonitrile) to the low pH columns. Each eluted fraction was desalted on the trap column after a ten times online dilution to pH 3 and subsequently separated on the analytical column; flow rate 250 nL/min, solvent A (0.1% formic acid in water) and solvent B (0.1% formic acid in acetonitrile), linear gradient 0 min, 99% A; 5 min, 93% A; 140 min, 65% A. The total run time was 180 min. The acquisition method was a TopN-MSMS where N was set to 12, meaning that the spectrometer acquires one Full MS spectrum, selects the 12 most intense peaks in this spectrum (singly charged precursors excluded) and makes a Full MS2 spectrum of each of these 12 compounds. The parameters for MS spectrum acquisition are: mass range from 400 to 1750 m/z, resolution of 70000, automated gain control (AGC) target of 1e6 or maximum injection time of 200 ms. The parameters for MS2 spectrum acquisition are: isolation window of 2.0 m/z, normalized collision energy (NCE) of 25, resolution of 17500, AGC target of 1e5 or maximum injection time of 50 ms, underfill ratio of 1.0%. The database searches were performed by the software MaxQuant version 1.6.0.16. Protein identifications were considered as significant if a protein was identified with at least two peptides, including at least one unique peptide. Peptide spectrum match (PSM) and protein false discovery rates (FDR) were both set at 0.01 (1%). Data normalization was performed using the LFQ algorithm ^2^. The minimum ratio count for LFQ was set at 2. The Perseus software (version 1.6.0.7) was used to perform further downstream statistical/bioinformatic analyses of the MaxQuant processing results. LFQ intensities were Log2 transformed for all statistical analysis. Only proteins identified and quantified in three *N* of at least one of the two groups (TM-GBM and SVZ-GBM cells) were considered for further analysis. Welch’s *t*-test finally allowed identification of proteins significantly differentially expressed between TM-GBM and SVZ-GBM cells.

## Western blot

Whole- cell lysates were prepared with a lysis buffer containing 500 mM NaCl, 50 mM TrisHCl, 1 mM EDTA, 1% NP40 and supplemented with 1X HALT^TM^ Protease and Phosphatase Inhibitor Cocktail EDTA-free (ThermoFisher). After a 10 min incubation on ice, protein lysates were further homogenized by ultrasonication then cell debris were removed by centrifugation (12 000 x g for 10 min). Protein concentrations were assessed using the Protein Assay Dye Reagent (Bio-Rad) based on the Bradford method. 30 μg of protein extracts were loaded and resolved on a homemade 10% acrylamide/bis-acrylamide gel (~ 30 min at 100 volts then 90 min at 120 volts) before being transferred (2h at 50 volts) onto a methanol activated PVDF membrane (Sigma-Aldrich). Membranes were then incubated one hour at room temperature in a blocking buffer (5% whole fat milk in TBS-Tween20) before being probed with primary antibodies overnight at 4°C. The primary antibodies used were the following: CD276 (Abcam, ab226256, 1/500), SOX2 (Cell Signaling, CS3579, 1/1 000), Nestin (Santa Cruz, 1/500), β-Actin- horseradish peroxidase (HRP) (Sigma-Aldrich, A3854, 1/10 000). Then, membranes were incubated with HRP-conjugated secondary antibodies for one hour at room temperature. Secondary antibodies used were the following: Goat anti-rabbit (Cell signaling, 7074, 1/2 000) and Horse anti-mouse (Cell signaling, 7076, 1/2 000). Finally, membranes were incubated with a chemiluminescent substrate (enhanced chemiluminescent (ECL), Life Technologies) for HRP, therefore generating a chemiluminescent signal detected with the ImageQuantTM LAS 4000 (GE Healthcare). Relative protein expression quantification was finally performed using ImageQuant TL Software (GE Healthcare).

## qPCR

Primers sequences used for B7-H3 and the housekeeping genes (GAPDH, 18S and β-Actin) mRNA amplification and quantification are the following: B7-H3: 5’-CTCTGCCTTCTCACCTCTTTG-3’ (forward primer), 5’-CCTTGAGGGAGGAACTTTATC-3’ (reverse primer). GAPDH: 5’-GGACTCATGACCACAGTCCAT-3’ (forward primer), 5’-GTTCAGCTCAGGGATGACCTT-3’ (reverse primer). 18S: 5’-CGATGCGGCGGCGTTATTC-3’ (forward primer), 5’-TCTGTCAATCCTGTCCGTGTCC-3’ (reverse primer). β-Actin: 5’-CGAGGCCCAGAGCAAGAGAG-3’ (forward primer), 5’-CGGTTGGCCTTAGGGTTCAG-3’ (reverse primer). Primers efficiency was validated before quantifying the relative expression of B7-H3 gene in relation to the housekeeping genes using the 2^-ΔΔCt^ comparative method.

## Immunofluorescence staining

The following primary antibodies were incubates at 4°C overnight: CD276 (Abcam, ab226256, 1/500), SOX2 (R&D system, MAB2018, 1/250), Nestin (Santa Cruz, Sc23927, 1/250), Iba1 (Abcam, Ab5076, 1/1 000), TMEM119 (Abcam, AMAB91528, ½ 500), PDGFRβ (R&D system, AF385, 1/250), CD31 (Agilent, MO82301-2, 1/250), human nuclei (Sigma-Aldrich, MAB1281, 1/250) and GFP (Abcam, ab13970, 1/500). Then, cells or tissues were incubated for 3h at room temperature with secondary antibodies conjugated with a fluorescent dye (Jackson Immuno-Research, diluted at 1:500). Finally, nuclei were labelled with a 4, 6-diamidino-2-phenylindole (DAPI) solution (Sigma-Aldrich, 1:10 000 in H_2_O) for 5 min. Images were acquired with a confocal Laser Scanning Biological Microscope (Olympus® Fluoview (FV) 1000) at a magnification specified for each experiment. The percentage of cell types in B7-H3 positive cells was quantified following acquisition of three photos of each specimen.

## Cell proliferation assay (EdU assay)

GBM cells were plated at 5 000 cells/well in a 96-well plate the day before the assay. As a control for proliferation, cells were treated for 16 h with a combination of Ara-C (2 µM, Acros Organics) and FUdR (10 µM, Acros Organics), two mitotic inhibitors. Cells were then treated with 10 µM of 5-Ethynyl-2’-deoxyuridine (5-EdU, Jena Bioscience) for 3 h. 5-EdU is a thymidine analogue which incorporates into DNA during cell division. The ethynyl groups present in EdU can be detected with Cu(I)-catalysed click chemistry (“*Click-it*” chemistry) in cells which underwent cell division. At the end of the experiment, GBM cells were fixed with 4% PFA for 10 min, washed and permeabilized with PBS-T, then were incubated 20 min with a staining solution containing 100 mM Tris pH 8.5, 2 mM CuSO_4_ (Sigma-Aldrich), 2 µM cy5 Azide (Jena Bioscience) and 100 mM L-Ascorbic acid (Sigma-Aldrich). Nuclei were counterstained 5 min with DAPI (1/10 000 in H_2_O). Cells were finally imaged with a confocal Laser Scanning Biological Microscope (Olympus® Fluoview (FV) 1000). Each condition was performed in four technical replicates and one field of each replicate was imaged. This experiment was performed three independent times (*N* = 3). Finally, cell proliferation rate was determined using the percentage of EdU positive cells (cy5 positive nuclei) relative to total cell number (DAPI positive nuclei).

## Cell viability assay (MTT assay)

Cells were plated at 5 000 GBM cells/well in a 96-well plate and allowed to attached overnight. Cells were then incubated for 3h at 37°C with 0.15 mg/ml of Thiazolyl Blue Tetrazolium Bromide (MTT, Sigma-Aldrich) diluted in DMEM serum free. Living cells can reduce MTT into purple crystals of formazan thereafter solubilized into Dimethylsulfoxyde (DMSO, Sigma). Directly after adding DMSO, absorbance was measured at 580 nm with a spectrophotometer (ThermoLab system Multiskan Ascent Platereader, Thermo Fisher Scientific). The absorbance is proportional to the number of living cells allowing cell viability measurement. Each condition was done in three technical replicates and repeated three independent times (*N* = 3).

## Apoptosis assay

GBM cells were treated with Temozolomide (TMZ, bio-techne), etoposide (Etop, bio-techne) or vehicle (DMSO) during 48 h. Cells in suspension (dead cells) and cells detached with Accutase were collected and at least 1x10^5^ cells were resuspended in 100 µl of Annexin V binding buffer containing 10 mM HEPES, 140 mM NaCl, and 2.5 mM CaCl2, pH 7.4. After addition of 5 μl of Annexin V conjugated with allophycocyanin (APC) (Fisher Scientific) and/or DAPI at a final concentration of 2.5 µg/ml, cells were incubated 15 min in the dark at room temperature, then were immediately analyzed with a BD LSRFortessa^TM^ (BD Bioscience) cell analyzer. Annexin V^-^/DAPI^-^ (Q4), Annexin V^+^/DAPI^–^(Q3), Annexin V^+^/DAPI^+^(Q2) and Annexin V^–^/DAPI^+^(Q1) cells correspond to living, early apoptotic, late apoptotic and necrotic cells respectively. Analysis were performed using FlowJo_V10® software. Graphs represent a percentage of Q2+Q3 (late and early apoptotic) cells relative to shNT OEctrl.

**Table S1: Additional information for patients from Rembrandt, TCGA and CGGA databases.**

| Grade (%) | | | |  |  |  |  |  |
| --- | --- | --- | --- | --- | --- | --- | --- | --- |
|  | | Sex (Male  /Female/*NA*)  (%) | Age (Year) (mean ± SD) | IDH (mut./  WT/*NA)*  (%) | II | III | IV | *NA* |
| **Rembrandt** | Astrocytoma | *NA* | *NA* | *NA* | 43.5 | 39.4 | 0 | 17.1 |
|  | Oligodendroglioma | *NA* | *NA* | *NA* | 44.8 | 34.3 | 0 | 20.9 |
|  | GBM | *NA* | *NA* | *NA* | 0 | 0 | 59.8 | 40.2 |
| **TCGA** | Astrocytoma | 49/38/13 | 42,1  ± 12,7 | 69.6/29.4  /1 | 30.4 | 59.8 | 0 | 9.8 |
|  | Oligoastrocytoma | 49/39/12 | 40,9  ± 13,6 | 87.7/12.3  /0 | 50 | 40.8 | 0 | 9.2 |
|  | Oligodendroglioma | 51/40/9 | 45,6  ± 13,8 | 88.5/11  /0.5 | 53.4 | 39.3 | 0 | 7.3 |
|  | GBM | 65/35/0 | 59,7  ± 13,5 | 6.6/90.8  /2.6 | 0 | 0 | 98 | 2 |
| **CGGA** | Astrocytoma | 64/36/0 | 38,5  ± 9,6 | 70.3/25.1  /4.6 | 100 | 0 | 0 | 0 |
|  | Oligoastrocytoma | 67/33/0 | 38,1  ± 9,7 | 44.4/0  /55.6 | 100 | 0 | 0 | 0 |
|  | Oligodendroglioma | 58/42/0 | 40,3  ± 9 | 92/4.5  /3.5 | 100 | 0 | 0 | 0 |
|  | Anaplastic Astrocytoma | 60/40/0 | 40,5  ± 11,6 | 58.4/39.7  /1.9 | 0 | 100 | 0 | 0 |
|  | Anaplastic Oligoastrocytoma | 33/67/0 | 40,3  ± 10,5 | 38.1/0  /61.9 | 0 | 100 | 0 | 0 |
|  | Anaplastic Oligodendroglioma | 46/54/0 | 42,6  ± 10,3 | 81.9/9.6  /8.5 | 0 | 100 | 0 | 0 |
|  | GBM | 61/39/0 | 48  ± 13,4 | 23.2/74.2  /2.6 | 0 | 0 | 100 | 0 |

*GBM: glioblastoma, IDH: isocitrate dehydrogenase, mut.: mutated, WT: wild-type, NA: data not available.*

**Table S2: Additional information for formalin-fixed paraffin-embedded glioblastoma and non-cancerous brain tissues.**

| **Patient’s**  **number** | **Pathology** | **Age** | **Sex** | ***IDH*** | **SOX-2** | **p53** | **EGFR** | **Ki67** | **MGMT** |
| --- | --- | --- | --- | --- | --- | --- | --- | --- | --- |
| BPTP18/01 | GBM | 77 | M | WT | NA | + | + | 5% | Met. |
| BPTP18/02 | GBM | 70 | M | WT | NA | + | + | 30% | Met. |
| BPTP18/03 | GBM | 46 | F | WT | NA | + | + | 30-40% | NA |
| BPTP18/04 | GBM | 63 | F | mut | NA | NA | + | 7-8% | Met. |
| BPTP18/05 | GBM | 70 | M | NA | NA | NA | NA | NA | NA |
| BPTP18/06 | GBM | 71 | M | WT | NA | + | + | 10% | Met. |
| Non-cancerous brain tissues | | | | | | | | | |
| BPTN18/07 | E | 43 | F |  |  |  |  |  |  |
| BPTN18/08 | E | 44 | F |  |  |  |  |  |  |
| BPTN18/09 | E | 40 | F |  |  |  |  |  |  |
| BPTN18/10 | NA | 47 | F |  |  |  |  |  |  |
| BPTN18/11 | NA | 30 | F |  |  |  |  |  |  |

*GBM: glioblastoma, E: epilepsy, M:male, F: female, IDH: isocitrate dehydrogenase, WT: wild-type, mut: mutated, EGFR: Epidermal Growth Factor Receptor, MGMT:* O*-6-Methylguanine-DNA Methyltransferase, Met.: methylated, NA: data not available.*

**Table S3: Additional information for fresh frozen newly diagnosed and recurrent glioblastoma and non-cancerous brain tissues.**

| **Patient’s**  **number** | **Patho-logy** | **Age** | **Sex** | ***IDH*** | **SOX-2** | **p53** | **EGFR** | **Ki67** | **MGMT** | **Therapy** |
| --- | --- | --- | --- | --- | --- | --- | --- | --- | --- | --- |
| 1.1 | ND | 69.0 | M | NA | + | 10% | - | 10-15% | Met. | Stupp |
| 1.2 | R | 69.4 | M | NA | + | 10% | - | 5-6% | Met. |  |
| 2.1 | ND | 63.0 | F | NA | + | <10% | + | 5% | Met. | Stupp |
| 2.2 | R | 63.5 | F | NA | + | - | + | 1-2% | Non-Met. |  |
| 3.1 | ND | 55.1 | M | NA | + | <10% | - | 10-15% | Non-Met. | Stupp |
| 3.2 | R | 56.8 | M | NA | + | <10% | + | 15-20% | Non-Met. |  |
| 4.1 | ND | 53.6 | F | NA | + | + | + | 25% | Non-Met. | Stupp |
| 4.2 | R | 54.1 | F | NA | + | + | - | 30% | Non-Met. |  |
| 5.1 | ND | 51.9 | F | NA | + | <10% | + | 25% | Met. | Stupp |
| 5.2 | R | 54.3 | F | NA | NA | <10% | + | 50% | Met. |  |
| 6.1 | ND | 51.8 | M | NA | + | <10% | + | 30% | Non-Met. | Stupp |
| 6.2 | R | 53.0 | M | WT | NA | <10% | + | 50% | Non-Met. |  |
| 7.1 | ND | 49.2 | M | WT | + | <10% | NA | 30% | Non-Met. | Stupp |
| 7.2 | R | 50.3 | M | NA | - | - | + | 30-40% | Non-Met. |  |
| 8.1 | ND | 45.0 | M | NA | NA | NA | + | 10-12% | Non-Met. | Stupp |
| 8.2 | R | 46.8 | M | NA | NA | <10% | + | 20-30% | Non-Met. |  |
| 9.1 | ND | 45.4 | F | NA | NA | 20-30% | + | 20-25% | Non-Met. | Stupp |
| 9.2 | R | 48.3 | F | NA | + | 15-20% | + | 30% | Met. |  |
| 10.1 | ND | 46.3 | M | NA | + | - | + | 25% | Non-Met. | Stupp |
| 10.2 | R | 47.2 | M | NA | + | - | + | 20% | Non-Met. |  |
| 11.1 | ND | 39.6 | M | WT | + | - | - | 20% | Non-Met. | Stupp |
| 11.2 | R | 42.5 | M | NA | NA | 10-15% | + | 10% | Non-Met. |  |
| Non-cancerous brain tissues | | | | | | | | | | |
| 12 | E | 44 | F |  | | | | | | |
| 13 | E | 40 | F |  |  |  |  |  |  |  |
| 14 | E | 33 | F |  |  |  |  |  |  |  |

*ND : newly diagnosed glioblastoma, R : glioblastoma recurrence, E: epilepsy, M:male, F: female, IDH: isocitrate dehydrogenase, WT: wild-type, EGFR: Epidermal Growth Factor Receptor, MGMT: O-6-Methylguanine-DNA Methyltransferase, Met.: methylated, Non-Met.: non-methylated, Stupp: Schema of therapy that comprises surgery followed by radiotherapy and chemotherapy using temozolomide. GBM: glioblastoma, NA: data not available.*

**Table S4: Additional information for fresh frozen glioblastoma and non-cancerous brain tissues.**

| **Patient’s**  **number** | **Patho-logy** | **Age** | **Sex** | ***IDH*** | **SOX-2** | **p53** | **EGFR** | **Ki67** | **MGMT** |
| --- | --- | --- | --- | --- | --- | --- | --- | --- | --- |
| BCTP19/01 MDG | GBM | 43 | F | NA | + | <10% | + | 30% | Non-Met. |
| BCTP19/02 MDG | GBM | 44 | F | WT | + | - | + | 5% | Met. |
| BCTP19/03 MDG | GBM | 35 | F | NA | NA | NA | NA | 50-60% | Non-Met. |
| BCTP19/04 MDG | GBM | 81 | F | WT | NA | 20% | + | 30-40% | Met. |
| BCTP19/05 MDG | GBM | 36 | F | NA | + | - | + | 10% | Non-Met. |
| BCTP19/06 MDG | GBM | 29 | M | NA | NA | NA | NA | NA | Met. |
| T19 | GBM | 57 | F | WT | NA | NA | + | 10-15% | NA |
| T20 | GBM | 49 | NA | WT | NA | - | + | NA | Met. |
| T22 | GBM | 68 | F | WT | NA | NA | + | 20% | Non-Met |
| T25 | GBM | 76 | M | WT | NA | NA | + | 30% | NA |
| T26 | GBM | 63 | M | WT | NA | NA | + | 10% | Met. |
| T28 | GBM | 58 | M | WT | NA | NA | + | 10-15% | NA |
| T29 | GBM | 82 | F | WT | NA | NA | + | 40% | Non-Met. |
| T30 | GBM | 62 | F | WT | NA | NA | + | 25-30% | Met. |
| Non-cancerous brain tissues | | | | | | | | | |
| BCTN18/07 NC | NA | 43 | F |  |  |  |  |  |  |
| BCTN18/08 NC | NA | 44 | F |  |  |  |  |  |  |
| BCTN18/09 NC | NA | 40 | F |  |  |  |  |  |  |
| BPTN19/10 MDG | HS | 33 | F |  |  |  |  |  |  |
| BPTN19/11 MDG | NA | 16 | M |  |  |  |  |  |  |
| BPTN19/12 MDG | ALS | 69 | F |  |  |  |  |  |  |
| NC1 | WM from P | 89 | F |  |  |  |  |  |  |
|  | GM from P |  |  |  |  |  |  |  |  |

*GBM: glioblastoma, ALS: amyotrophic lateral sclerosis, HS: hippocampal sclerosis, WM: white matter, GM: grey matter, P: Parkinson disease, M: male, F: female, IDH: isocitrate dehydrogenase, WT: wild-type, EGFR: epidermal growth factor receptor, MGMT: O-6-Methylguanine-DNA Methyltransferase, Met.: methylated, Non-Met.: non-methylated, NA: data not available.*

**Table S5: Additional information for primary glioblastoma cells.**

| **Cells** | **Pathology** | **Age** | **Sex** | ***IDH*** | **Ki67** | **MGMT** |
| --- | --- | --- | --- | --- | --- | --- |
| GB138 | GBM | 48 | F | WT | NA | Met. |
| T08 | GBM | 57 | F | WT | 30% | Non-Met. |
| T018 | GBM | 61 | M | WT | 10% | Non-Met. |

*GBM: glioblastoma, M: male, F: female, IDH: isocitrate dehydrogenase, WT: wild-type, MGMT: O-6-Methylguanine-DNA Methyltransferase, Met.: methylated. and Non-Met.: non-methylated, NA: data not available.*

**Table S6: shRNA sequences used to target human CD276 gene or as control (non-target, NT).**

| **Origin** | **Target gene** | **shRNA sequences** |
| --- | --- | --- |
| Human | *CD276* | ATC CTGCCTGCTG CCTTATTTCT CGAGAAATAA GGCAGCAGGC AGGA |
|  | *CD276* | TCC ATTCAGTTGA TGTTTATTCTCGAGAATAAA CATCAACTGA ATGGA |
|  | *CD276* | CT CTTGCTCTAG CCTTAATACT CGAGTATTAA GGCTAGAGCA AGAGG |
|  | *CD276* | CT GGGAGACAGA CAACTAACCT CGAGGTTAGT TGTCTGTCTC CCAGC |
|  | *CD276* | TTT CTCCAATGGC CGTGATACCT CGAGGTATCA CGGCCATTGG AGAAA |
|  | *NT* | GCCT AAGGTTAAGT CGCCCTCGCT CGAGCGAGGG CGACTTAACC TTAGG |

**Table S7: cDNA sequences used to over-express CD276 gene coding for the human 2IgB7-H3 or 4IgB7-H3 isoforms.**

| **Origin** | **gene** | **Variant** | **cDNA sequences (5’-3’)** |
| --- | --- | --- | --- |
| Human | *CD276* | 2Ig | AT GCTGCGTCGG CGGGGCAGCC CTGGCATGGG TGTGCATGTG GGTGCAGCCC TGGGAGCACT GTGGTTCTGC CTCACAGGAG CCCTGGAGGT CCAGGTCCCT GAAGACCCAG TGGTGGCACT GGTGGGCACC GATGCCACCC TGTGCTGCTC CTTCTCCCCT GAGCCTGGCT TCAGCCTGGC ACAGCTCAAC CTCATCTGGC AGCTGACAGA TACCAAACAG CTGGTGCACA GCTTTGCTGA GGGCCAGGAC CAGGGCAGCG CCTATGCCAA CCGCACGGCC CTCTTCCCGG ACCTGCTGGC ACAGGGCAAC GCATCCCTGA GGCTGCAGCG CGTGCGTGTG GCGGACGAGG GCAGCTTCAC CTGCTTCGTG AGCATCCGGG ATTTCGGCAG CGCTGCCGTC AGCCTGCAGG TGGCCGCTCC CTACTCGAAG CCCAGCATGA CCCTGGAGCC CAACAAGGAC CTGCGGCCAG GGGACACGGT GACCATCACG TGCTCCAGCT ACCGGGGCTA CCCTGAGGCT GAGGTGTTCT GGCAGGATGG GCAGGGTGTG CCCCTGACTG GCAACGTGAC CACGTCGCAG ATGGCCAACG AGCAGGGCTT GTTTGATGTG CACAGCGTCC TGCGGGTGGT GCTGGGTGCG AATGGCACCT ACAGCTGCCT GGTGCGCAAC CCCGTGCTGC AGCAGGATGC GCACGGCTCT GTCACCATCA CAGGGCAGCC TATGACATTC CCCCCAGAGG CCCTGTGGGT GACCGTGGGG CTGTCTGTCT GTCTCATTGC ACTGCTGGTG GCCCTGGCTT TCGTGTGCTG GAGAAAGATC AAACAGAGCT GTGAGGAGGA GAATGCAGGA GCTGAGGACC AGGATGGGGA GGGAGAAGGC TCCAAGACAG CCCTGCAGCC TCTGAAACAC TCTGACAGCA AAGAAGATGA TGGACAAGAA ATAGCCTGA |
|  | *CD276* | 4Ig | AT GCTGCGTCGG CGGGGCAGCC CTGGCATGGG TGTGCATGTG GGTGCAGCCC TGGGAGCACT GTGGTTCTGC CTCACAGGAG CCCTGGAGGT CCAGGTCCCT GAAGACCCAG TGGTGGCACT GGTGGGCACC GATGCCACCC TGTGCTGCTC CTTCTCCCCT GAGCCTGGCT TCAGCCTGGC ACAGCTCAAC CTCATCTGGC AGCTGACAGA TACCAAACAG CTGGTGCACA GCTTTGCTGA GGGCCAGGAC CAGGGCAGCG CCTATGCCAA CCGCACGGCC CTCTTCCCGG ACCTGCTGGC ACAGGGCAAC GCATCCCTGA GGCTGCAGCG CGTGCGTGTG GCGGACGAGG GCAGCTTCAC CTGCTTCGTG AGCATCCGGG ATTTCGGCAG CGCTGCCGTC AGCCTGCAGG TGGCCGCTCC CTACTCGAAG CCCAGCATGA CCCTGGAGCC CAACAAGGAC CTGCGGCCAG GGGACACGGT GACCATCACG TGCTCCAGCT ACCAGGGCTA CCCTGAGGCT GAGGTGTTCT GGCAGGATGG GCAGGGTGTG CCCCTGACTG GCAACGTGAC CACGTCGCAG ATGGCCAACG AGCAGGGCTT GTTTGATGTG CACAGCATCC TGCGGGTGGT GCTGGGTGCA AATGGCACCT ACAGCTGCCT GGTGCGCAAC CCCGTGCTGC AGCAGGATGC GCACAGCTCT GTCACCATCA CACCCCAGAG AAGCCCCACA GGAGCCGTGG AGGTCCAGGT CCCTGAGGAC CCGGTGGTGG CCCTAGTGGG CACCGATGCC ACCCTGCGCT GCTCCTTCTC CCCCGAGCCT GGCTTCAGCC TGGCACAGCT CAACCTCATC TGGCAGCTGA CAGACACCAA ACAGCTGGTG CACAGTTTCA CCGAAGGCCG GGACCAGGGC AGCGCCTATG CCAACCGCAC GGCCCTCTTC CCGGACCTGC TGGCACAAGG CAATGCATCC CTGAGGCTGC AGCGCGTGCG TGTGGCGGAC GAGGGCAGCT TCACCTGCTT CGTGAGCATC CGGGATTTCG GCAGCGCTGC CGTCAGCCTG CAGGTGGCCG CTCCCTACTC GAAGCCCAGC ATGACCCTGG AGCCCAACAA GGACCTGCGG CCAGGGGACA CGGTGACCAT CACGTGCTCC AGCTACCGGG GCTACCCTGA GGCTGAGGTG TTCTGGCAGG ATGGGCAGGG TGTGCCCCTG ACTGGCAACG TGACCACGTC GCAGATGGCC AACGAGCAGG GCTTGTTTGA TGTGCACAGC GTCCTGCGGG TGGTGCTGGG TGCGAATGGC ACCTACAGCT GCCTGGTGCG CAACCCCGTG CTGCAGCAGG ATGCGCACGG CTCTGTCACC ATCACAGGGC AGCCTATGAC ATTCCCCCCA GAGGCCCTGT GGGTGACCGT GGGGCTGTCT GTCTGTCTCA TTGCACTGCT GGTGGCCCTG GCTTTCGTGT GCTGGAGAAA GATCAAACAG AGCTGTGAGG AGGAGAATGC AGGAGCTGAG GACCAGGATG GGGAGGGAGA AGGCTCCAAG ACAGCCCTGC AGCCTCTGAA ACACTCTGAC AGCAAAGAAG ATGATGGACA AGAAATAGCC TGA |
