# Supplementary material for: The expression of B7-H3 isoforms in newly diagnosed glioblastoma and recurrence and their functional role
Source: Acta Neuropathol Commun. 2021 Apr 1;9:59. doi: 10.1186/s40478-021-01167-w (PMC8017683; doi:10.1186/s40478-021-01167-w)
Supplement: Supplementary file 2 — Additional file 2. Additional_file_2_figures_Acta_Neuropath._Comm_review. This file contains Supplementary Figures and legends S1–S6. [file 40478_2021_1167_MOESM2_ESM.docx]

# Additional file 2


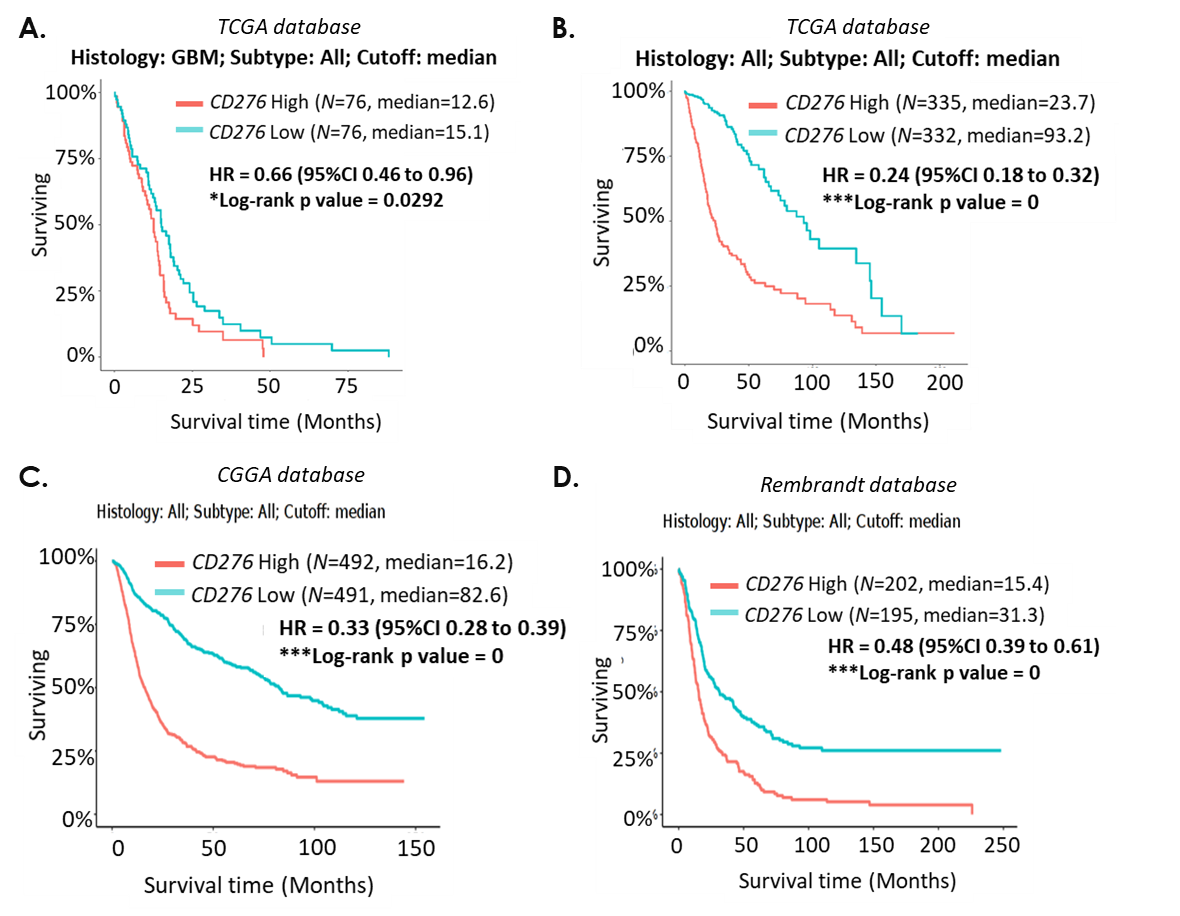


**Fig. S1: *CD276* mRNA expression is an independent prognosis factor in GBM and all glioma patients. A.** Data from The Cancer Genome Atlas (TCGA) database in GBM patients expressing high (*N* = 76, red) or low (*N* = 76, blue) *CD276* mRNA level **B.** Data from TCGA database in all glioma patients expressing high (*N* = 335, red) or low (*N* = 332, blue) *CD276* mRNA level. **C.** Data from the Chinese Glioma Genome Atlas (CGGA) database in glioma patients expressing high (*N* = 492, red) or low (*N* = 491, blue) *CD276* mRNA level. **D.** Data from REpository for Molecular BRAin Neoplasia DaTa (REMBRANDT) database in glioma patients expressing high (*N* = 202, red) or low (*N* = 195, blue) *CD276* mRNA level. Data were analyzed using GlioVis, an online data visualization and analysis tool. Data represent the percentage of surviving patients over time (Months). Hazard Ration (HR) represents the risk of death overtime and is associated with 95% confidence interval (95% CI). * p<0.05, *** p<0.001 (Log-rank test).


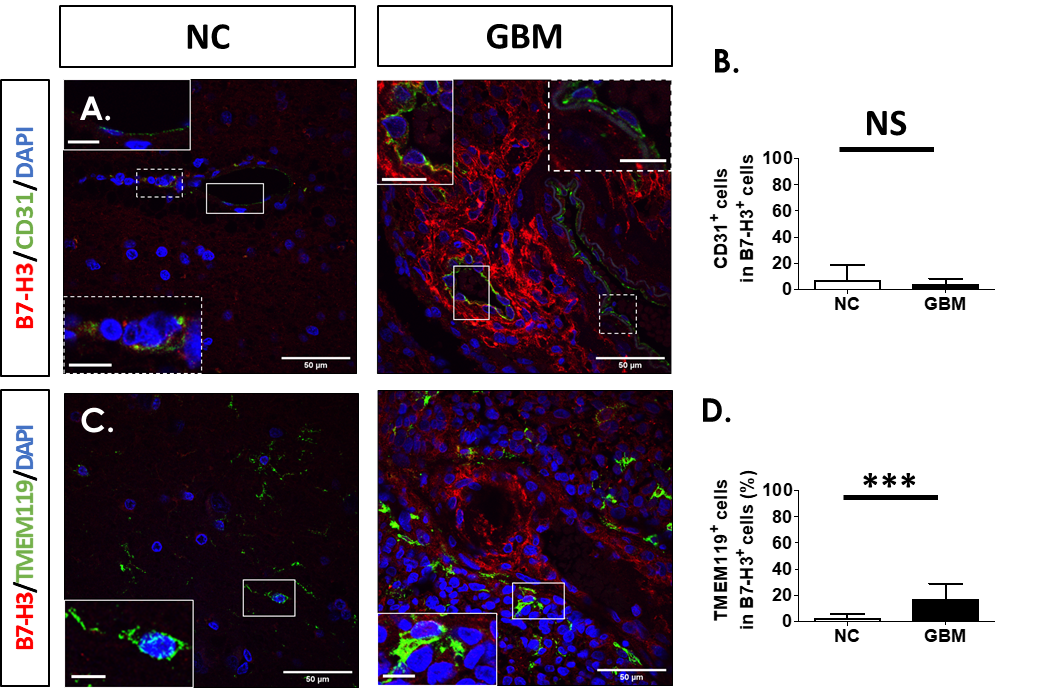


**Fig. S2: Proportion of cell type expressing B7-H3 in human non-cancerous brain *vs* GBM tissues.** Representative immunofluorescent staining in non-cancerous (NC) brain (*N* = 5) *vs* GBM (*N* = 6) tissues expressing B7-H3 (red) and: **A.** CD31 and **C.** TMEM119 in green and counterstained with DAPI (blue). Major scale bar = 50 µm and scale bar for enlarged images = 10 µm.  **B.** and **D.** Percentage of each cell type; CD31 and TMEM119 respectively in B7-H3 positive cells in NC brain (*N* = 5) *vs* GBM (*N* = 6) tissues. Three photos of each specimen were taken for quantification. Graphs are mean ± SD with NS = not significant and ***<0.001 (t-test).


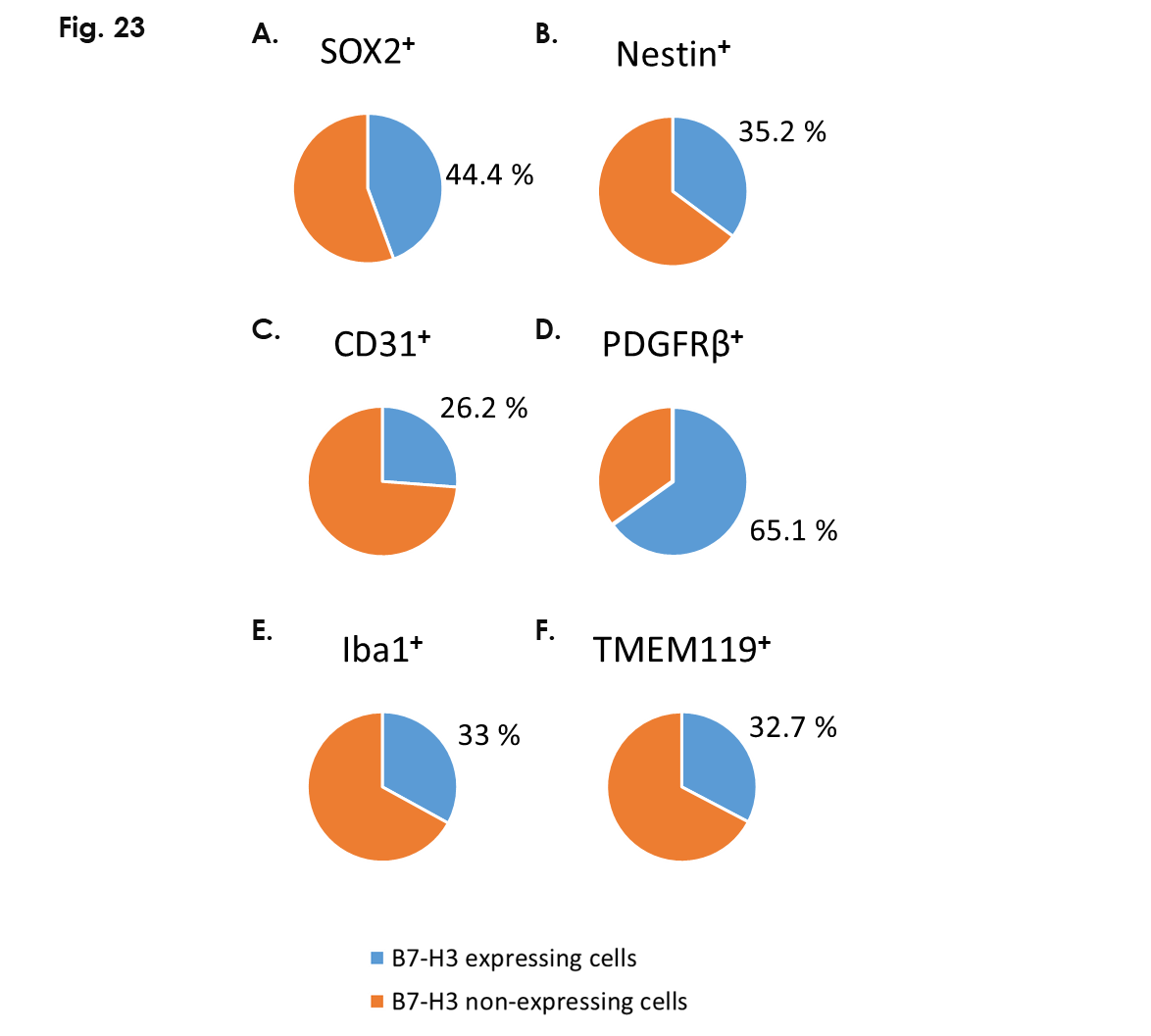


**Fig. S3: Proportion of B7-H3 expression among various cell types in glioblastoma (GBM) tissues. A - F.** Percentage of B7-H3 positive (blue) or negative (orange) cells in SOX2, Nestin, CD31, PDGFRβ, Iba1 or TMEM119 positive cells, respectively.


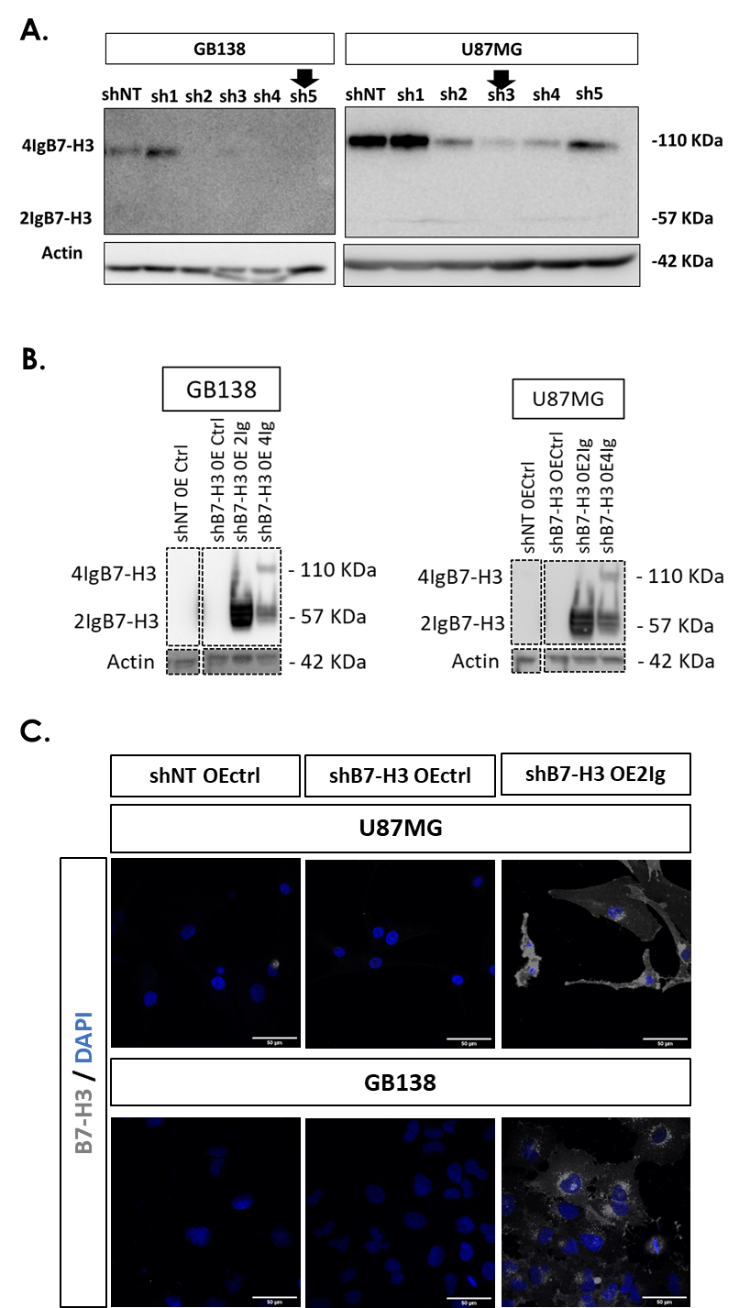


**Fig. S4: GBM cell model of B7-H3 decreased and/or increased expression.** Representative image of a western blot analysis of B7-H3 (4IgB7-H3: 110kDa; 2IgB7-H3: 57kDa) and Actin (42kDa) signals in proteins extracted from **A.** U87MG and GB138 GBM cells (*N* = 3 for each cell type) transduced with short hairpin RNA (shRNA) against the expression of B7-H3 (each shRNA were named with numbers) and compared to shRNA non-target (shNT). Black arrows indicate the shRNA designed to decrease B7-H3 expression in each cell type. **B.** U87MG and GB138 GBM cells (*N* = 3 for each cell type) transduced with shRNA against B7-H3 or shNT and vector over-expressing (OE) control (OEctrl), 2IgB7-H3 (OE2Ig) or 4IgB7-H3 (OE4Ig). **C.** Representative immunofluorescent staining of B7-H3 expression (grey) in U87MG and GB138 GBM cells modified as described in B. Nuclei were counterstained with DAPI (blue). Scale bar = 50 µm.


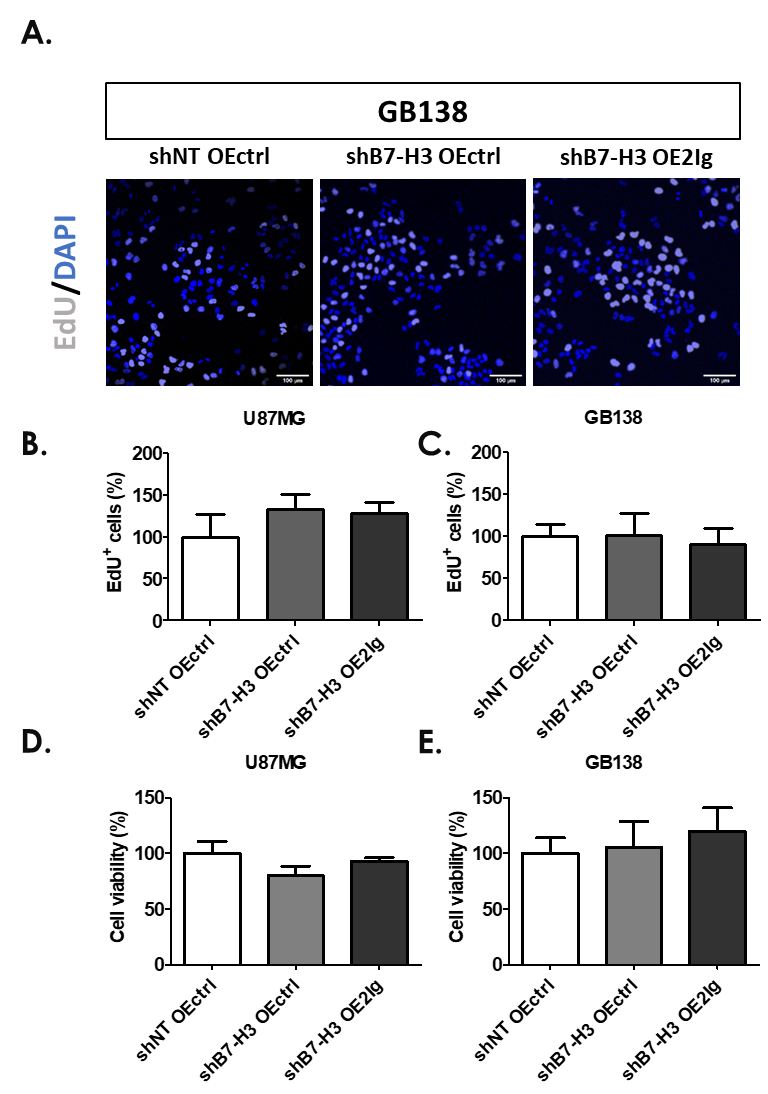


**Fig. S5: B7-H3 expression does not modify DNA replication before cell division nor viability of GBM cells. A.** Representative images of EdU staining (Grey) in GB138 human GBM cells transduced with short hairpin RNA (shRNA) against B7-H3 (shB7-H3) or non-target (shNT) and vector over-expressing (OE) 2IgB7-H3 (OE2Ig) or control (OEctrl). Nuclei were counterstained with DAPI (blue). Scale bar = 100 µm. **B.** and **C.** Graphs quantifying the number of EdU-positive U87MG and GB138 cells transduced with shRNA and over-expressing vector as described in **A.** Each EdU assay was performed in four technical replicate with an image took in each replicate and repeated three times. Numbers of EdU incorporating nuclei of different groups were normalized with the value from the shNT OEctrl group, expressed as a percentage and was shown as mean values ± SD (*N* = 3 for each cell type). **D.** and **E.** Graphs quantifying the cell viability by MTT assay in U87MG and GB138 GBM cells treated as described in **A.** Each cell viability assay was performed in triplicate and repeated three times. The cell viability of different groups were normalized with the value from the shNT OEctrl group, expressed as a percentage and was shown as mean values ± SD (*N* = 3 for each cell type).


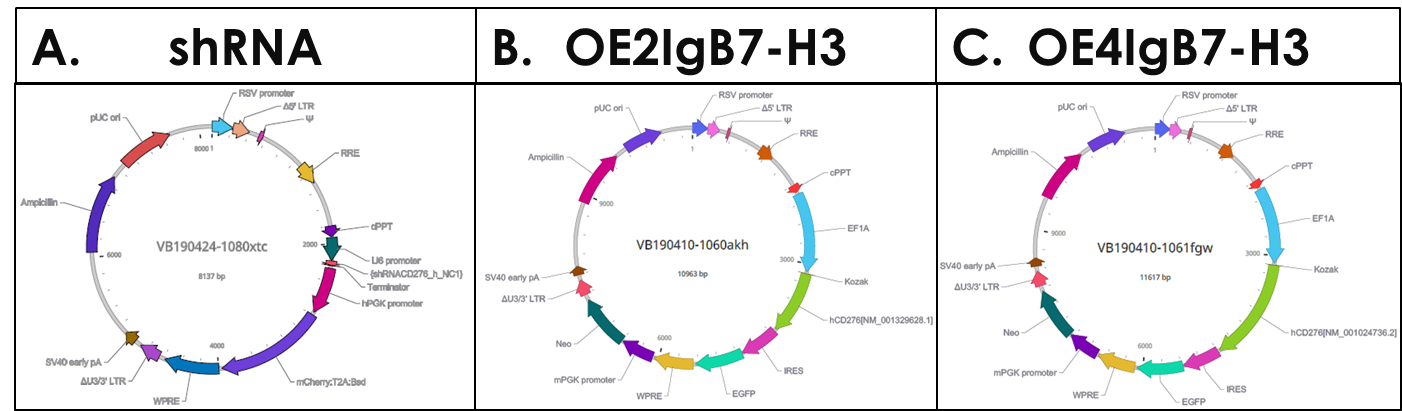


**Fig. S6: Vector maps for: A.** shRNA, **B.** OE2IgB7-H3 and **C.** OE4IgB7-H3.
